# A Review of Macrocycles Applied in Electrochemical Energy Storge and Conversion

**DOI:** 10.3390/molecules29112522

**Published:** 2024-05-27

**Authors:** Qijian Zhu, Danfei Fu, Qing Ji, Zhongjie Yang

**Affiliations:** 1 Department of Resources and Environment, Moutai Institute, Renhuai 564500, China; zhuqijian@mtxy.edu.cn; 2 School of Chemistry and Materials, Guizhou Normal University, Guiyang 550025, China; danfeifu@163.com

**Keywords:** macrocycles, electrochemical energy storge, battery, supercapacitor

## Abstract

Macrocycles composed of diverse aromatic or nonaromatic structures, such as cyclodextrins (CDs), calixarenes (CAs), cucurbiturils (CBs), and pillararenes (PAs), have garnered significant attention due to their inherent advantages of possessing cavity structures, unique functional groups, and facile modification. Due to these distinctive features enabling them to facilitate ion insertion and extraction, form crosslinked porous structures, offer multiple redox-active sites, and engage in host–guest interactions, macrocycles have made huge contributions to electrochemical energy storage and conversion (EES/EEC). Here, we have summarized the recent advancements and challenges in the utilization of CDs, CAs, CBs, and PAs as well as other novel macrocycles applied in EES/EEC devices. The molecular structure, properties, and modification strategies are discussed along with the corresponding energy density, specific capacity, and cycling life properties in detail. Finally, crucial limitations and future research directions pertaining to these macrocycles in electrochemical energy storage and conversion are addressed. It is hoped that this review is able to inspire interest and enthusiasm in researchers to investigate macrocycles and promote their applications in EES/EEC.

## 1. Introduction

**Figure 1:**
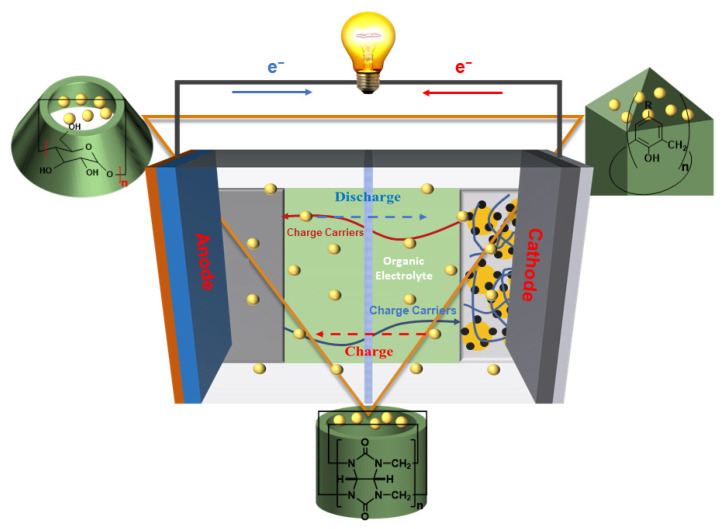
Illustration of the mechanism in EES/EEC devices.

The current challenges arising from the increasing demand for continuous energy supply and the scarcity of fossil fuels have necessitated the implementation of intelligent approaches to address these issues. This necessitates the application of sustainable energy sources, such as solar power, wind energy, tidal power, and geothermal resources. It is crucial to explore reliable and efficient devices for storing and converting electrochemical energy (EES/EEC), which plays a significant role in achieving sustainable solutions [1,2,3,4]. Rechargeable batteries and supercapacitors play major roles in energy storage and conversion devices [3,5,6,7,8,9,10,11,12,13]; the working mechanism of these devices is illustrated in Figure 1. Moreover, tremendous efforts have been devoted to advancing these high-performance energy storage and conversion devices, aiming for enhanced capacity, power density, and cycle life [14,15,16,17,18,19,20,21,22,23]. For instance, the utilization of lithium-ion batteries (LIBs) in our daily lives has significantly increased since the 1990s, with a notable achievement of achieving a high energy density of approximately 250 Wh kg^−1^ with an extended cycling life more than 1000 cycles [24,25,26,27]. Due to their exceptional theoretical specific capacity reaching up to 1675 mAh g^−1^ and an impressive energy density of 2600 Wh kg^−1^, lithium-S batteries (LSBs) have garnered considerable attention [28,29]. The alternatives such as sodium-ion batteries (SIBs) [8,9,30,31], potassium-ion batteries (PIBs) [32,33], magnesium-ion batteries (MIBs) [34,35,36], and zinc-ion batteries (ZIBs) have been reported to exhibit exceptional energy storage performance [37,38,39,40]. Furthermore, organic batteries have been extensively studied owing to their cost-effectiveness, recyclability, environmental friendliness, easy molecular design, and flexibility [41,42,43,44]. Despite these excellent achievements and advantages, there is an urgent need for rechargeable batteries and supercapacitors with significantly enhanced performance in order to meet the increasing energy demands of society. Generally, the performance of rechargeable batteries and supercapacitors severely depends on the key components of these energy storage devices, such as electrodes, electrolytes, separators, and current collectors [7,12]. Despite the impressive successes reported through extensive research, there still exist harder challenges in designing reasonable materials that primarily determine the performance of rechargeable batteries and supercapacitors. Compared to liquid LIBs, the transport of Li^+^ and safety concerns continue to pose obstacles for further advancements in LIB technology [45]. Solid-state LIBs, with their focus on solid-state electrolytes, have attracted significant interest for their ability to improve safety and enhance cycling performance [46,47]. However, the inherent poor solid–solid contact in solid-state LIBs poses significant challenges, leading to substantial electrochemical polarization, inferior performance, and limited Li^+^ conductivity [45,48,49]. Although lithium-sulfur batteries (LSBs) possess remarkable theoretical energy density, environmental friendliness, prospective reserves, and low cost, their electrochemical performance is severely limited by the low conductivity and shuttle of intermediate lithium polysulfide in the sulfur cathode as well as the instability of the anode/electrolyte interphase [45,49,50]. The main obstacles to the practical application of energy storage and conversion devices lie in their key components, which largely determine their development. In recent decades, a multitude of advanced functional materials, such as metal oxides [49,51], metal organic frameworks (MOFs) [52,53], covalent organic frameworks (COFs) [21], graphene [11,13], and polymers [54,55], have emerged as crucial components in the pursuit of batteries and cells with high energy density, high capacity, longer cycling performance, high rate, low cost, and stability.

**Figure 2:**
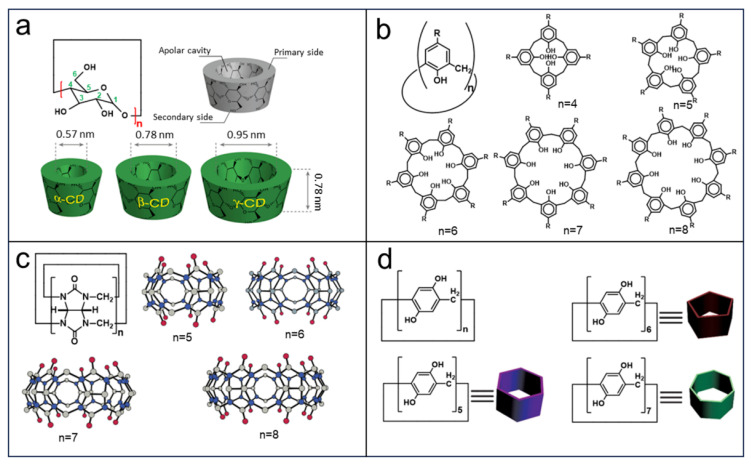
(**a**) Functional structural illustration of α-CD (*n* = 6), β-CD (*n* = 7), and γ-CD (*n* = 8). Bottom: the cavity structures with differential diameters’ corresponding CDs. Reprinted with permission from [60]. Copyright American Chemical Society, 2014. (**b**) Molecular structures of calix[n]arenes, *n* = 4–8. Reprinted with permission from [62]. Copyright Springer, 2005. (**c**) Molecular structures and X-ray crystal structures of cucurbit[n]urils, *n* = 5–8 [63,64]. Reprinted with permission from [62,63]. Copyright American Chemical Society, 2000 and 2003. (**d**) Chemical structures and schematic representations of pillar[*n*]arene, *n* = 5–7. Reprinted with permission from [65]. Copyright Wiley, 2013.

Macrocycles have received extensive attention due to the porosity and functional groups since these cycle molecules were widely applied in drug design [56], playing an important role in supramolecular chemistry. Indeed, cyclodextrins (CDs), calixarenes (CAs), cucurbiturils (CBs), and pillararenes (PAs), as well-established macrocycles, have demonstrated great potential in host–guest recognition and sensor applications [57,58,59], owing to their unique pore structures and modifiable functional group. CDs are polysaccharides produced through enzymatic hydrolysis of amylum, consisting of 6~8 D(+)-glucose units which are linked by α-1,4-linkages (Figure 2a), recorded as α-, β-, or γ-CD, respectively. The cage-like structure inherent in the molecular skeleton endows these macrocycles with the advantages of favorable ion conductivity. In addition, the abundant hydroxyl groups render them easily modified toward special functional frameworks [60]. CAs are the products of the condensation between *p*-substituted phenols and formaldehyde with base induced (Figure 2b), having an inherent macrocyclic core which can be available in a variety sizes, allowing for easy selective functionalization [61,62]. CBs are produced by combining glycoluril with an overabundance of formaldehyde via a condensation reaction, typically consisting of 5~8 or 10 glycoluril units named cucurbit[*n*]uril (Figure 2c) [63,64]. Notably, PAs possess pillar-shaped structures with redox-active site and electron-rich cavities (Figure 2d). Similar to cyclodextrins, pillararenes can easily be functionalized with bulk groups to form rigid chemical structures and exhibit selective binding towards guest molecules [65]. These distinctive characteristics of the aforementioned four categories of macrocycles make them highly applicable in EES/EEC. 

In the past few decades, macrocycles (including CDs, CAs, CBs, PAs, etc.) applied in EES/EEC have been extensively investigated. (a) Due to the special intrinsic cavity structure, these macrocycles can be designed as electrode and electrolyte materials, in terms of improving the performance of batteries and supercapacitors by impeding the ion diffusion and improving ion conductivity in electrodes and electrolytes, respectively [66,67]. Furthermore, the cavity structure serves as a template for preparing other porous structural materials that exhibit improved ion conductivity and stability [68]. (b) Quinones, as redox sites in macrocycles, possess more than eight carbonyl groups endowing these macrocycles with a high capacity due to the combination between the carbonyl group and lithium ion with no steric hindrance [69]. (c) The feature of easy functionalization of macrocycles makes them straightforward to synthesize crosslink frameworks or graft to other functional materials (including CNTs, GO, and polymers, etc.) resulting in better pore size and improved ion conductivity [70]. 

Due to the significant physical and chemical properties as well as the potential applicability, there have been a limited number of excellent reviews on macrocycles applied in EES/EEC. Desoky et al. provide a comprehensive overview of CD-based materials utilized in battery components, including binders, electrolytes, and separators in 2023 [66]. Zhang reviewed the applications of three types of quinone-based CAs and PAs in LIBs, SIBs, ZIBs, and MIBs [71]. However, almost no reviews focus on all four macrocycles of CDs, CAs, CBs, and PAs applied in EES/EEC. Hence, this comprehensive review mainly focuses on CDs-, CAs-, CBs, and PAs-based materials, aiming to provide a thorough and up-to-date overview of these macrocycles in outstanding EES/EEC applications. Firstly, the utilization of CDs, CAs, CBs, Pas, and other representative macrocycles in different energy storage devices, such as LIBs and alternative metal ion batteries as well as supercapacitors, will be comprehensively reviewed and compared. Furthermore, the correlation between the modification strategies and electrochemical performance in batteries or supercapacitors will be thoroughly discussed. Finally, we extensively discuss the current challenges and instructive study suggestions for the application of macrocycles as electroactive materials for advanced EES/EEC devices. We hope this comprehensive review will serve as a valuable reference to inspire the interest and enthusiasm of researchers in investigating macrocycles and promote their applications in EES/EEC devices.

## 2. CDs in Electrochemical Energy Storage and Conversion

CDs are derived from the enzymatic breakdown of decomposed starch, resulting in the production of synthetic products and are composed of 6–8 D(+)-glucose units, which are linked by α-1,4-linkages, abbreviated as α-, β-, or γ-CD, respectively. The unique structure enables CDs to be particularly practical. On the one hand, CDs are cage molecules with a dimensionally stable hydrophobic cavity within the molecule structure providing the capability of trapping or encapsulating guest molecules by host–guest interaction [60,66], leading to a modification and/or improvement in the physical, chemical, and/or biological natures of guest molecules [60]. On the other hand, there are dozens of hydroxyl groups in each molecule of CDs, leading to easy modification through chemical reactions. These features endow CDs with great practical applications in many sectors, especially food, medicine, chemistry, chromatography, biotechnology, etc. [72,73,74,75,76]. Additionally, the host–guest interaction and easy modification have been used to prepare organic and/or inorganic battery components, such as electrodes, electrolytes, and binders.

### 2.1. CDs in LIBs

**Figure 3:**
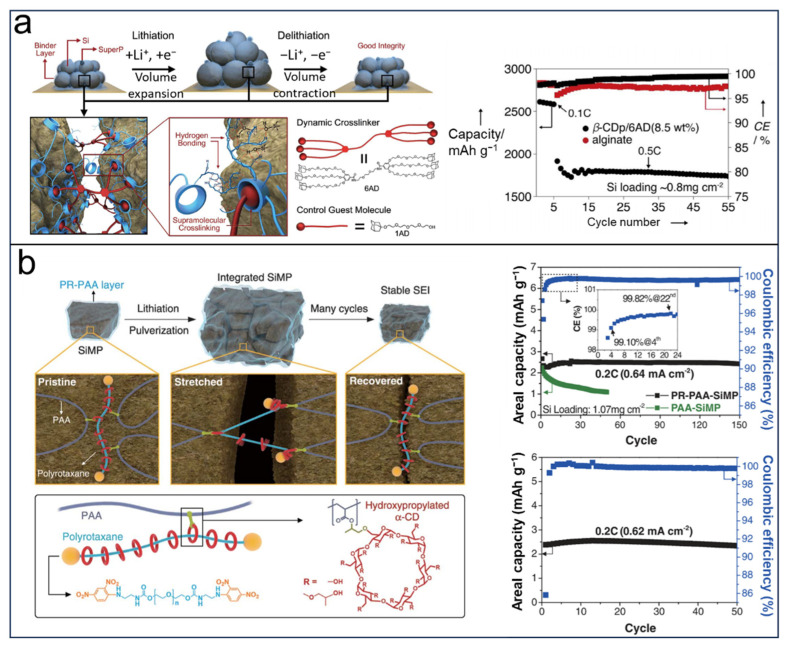
New binding mechanisms of CDs-based binders in lithium ion batteries: (**a**) Working mechanism of dynamic crosslinking of β-CDp and 6AD (left) and cycling performance. Reprinted with permission from [80]. Copyright American Chemical Society, 2015. (**b**) Graphical representation of the operation of PR-PAA binder and cycling performance. Reprinted with permission from [82]. Copyright Science.org, 2017.

LIBs have shown increasing fatigue in terms of the huge renewable energy demand [45]. Silicon (Si) anodes are one of the most potential candidates which have exceptionally excellent gravimetric capacities (4200 mAh g^−1^) for meeting this issue. However, the electrode’s structural collapse caused by volume expansion and its significant impact on cycling performance pose obstacles to the advancement of Si anodes [77,78,79]. Polymeric binder has proved to be a key role in maintaining electrode structure and consequently initial capacity [80,81]. Compared with common polymer binders, CDs have stable hydrophobic cavities that can create a host–guest interaction resulting in new binding mechanisms. For instance, Choi and Coskun et al. [80] reported that CDs-based polymer binders by supramolecular crosslinking, hyperbranched features of both β-cyclodextrin polymers (β-CDp), and the supramolecular crosslinker leading to the incorporation of six adamantane units can result in a host–guest interaction causing “dynamic crosslinking” (Figure 3a). The host–guest interactions provide the crosslinking system with the capacity to reversibly restore broken linkages among the polymer chains during the cycling process and to introduce a self-healing effect in addition to maintaining the interactions between Si and the binder, as well as preserving the film integrity throughout the charging and discharging cycles. Reaching a 90% capacity retention after 150 cycles at a rate of 0.5 C with a silicon loading of 0.8 mg cm^−2^, the excellent cycling performance observed can be attributed to the interaction between β-CDp as the host and guest components. In 2017, the same group proposed a novel concept of groundbreaking elastic crosslinking [82]. In this work, the linkage between poly-(acrylic acid) (PAA) and α-CDs-based pseudo-rotaxanes (PRs) generated a necklace-like and interlocked network marked as PR-PAA (Figure 3b). This strategy makes PR-PAA highly stretchable and elastic, resulting in being able to withstand significant strains (up to 390%) and excellent stress–strain recovery properties, which are superior to PAA. The key factor of the enhanced mechanical strength is owing to the feature that threaded CDs can skillfully slide along the polymer chains.

**Figure 4:**
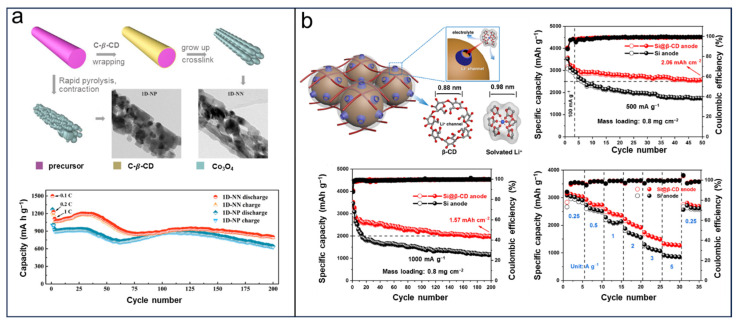
CDs-based binders for lithium-ion diffusion: (**a**) The formation illustration and cycling performance of 1D nanonet. Reprinted with permission from [88]. Copyright Elsevier, 2017. (**b**) Schematic representations of the Si@β-CD electrode and cycling performance. Reprinted with permission from [89]. Copyright Wiley, 2021.

Although polymer binders have proven to be an essential ingredient in high-capacity metal anodes, introducing a binder can influence the structure of the solid electrolyte interphase, leading to a great impact on electrochemical performance [83,84,85,86,87]. Fortunately, CDs-based binders were reported to be proficient in increasing Li^+^ transport channels and solving this crack. Hu et al. [88] reported enhanced-rate-performance LIBs using 1D Co_3_O_4_ nanonets as the electrode, in which the independent nanoparticles can be interconnected by a C-β-CD crosslinking agent (Figure 4a). The enhanced rate performance is attributed to the interconnection mechanism of a 1D nanonet structure, which facilitates the transport of Li+ ions. The anode, composed of a 1D nanonet based on C-β-CD, demonstrates an impressive rate capacity of approximately 982 mAh g^−1^ when discharged at a current density of 2000 mA g^−1^.

**Figure 5:**
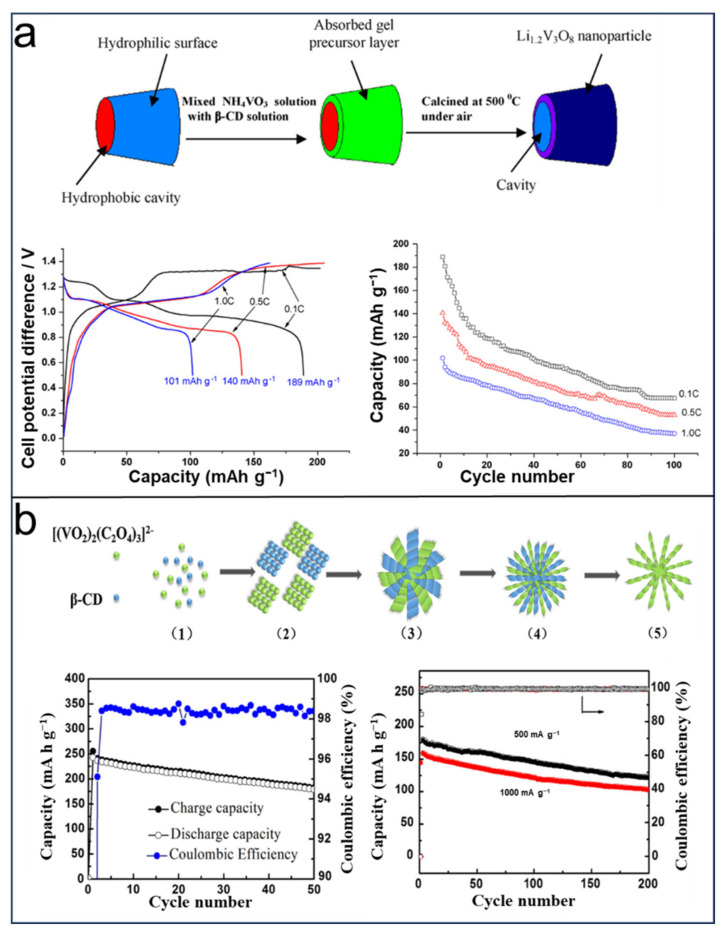
Schematic illustration for using β-CD as templates: (**a**) fabrication of macaroni-like Li_1.2_V_3_O_8_ nanoparticle and electrochemical performance. Reprinted with permission from [68]. Copyright Elsevier, 2010. (**b**) Fabrication and cyclic performance of 3D NH_4_V_4_O_10_. Reprinted with permission from [95]. Copyright Springer, 2018.

In 2021, Liu et al. designed a Li^+^ diffusion channel using β-CD to stabilize a Si anode [89]. As shown in Figure 4b, the dimensions of the microscopic channels for molecules within β-CD and the solvated lithium ions measure 0.88 nm and 0.98 nm, correspondingly. The approximately comparable diameters can promote the Li^+^ transport and filter out the solvent molecules. The stabilized silicon anode exhibited a remarkable reversible capacity of 2562 mAh g^−1^ at a current density of 500 mA g^−1^ after undergoing 50 cycles. Furthermore, it maintained a high capacity of 1944 mAh g^−1^ even after enduring 200 cycles at a higher current density of 1 A g^−1^. This innovative approach presents a novel strategy for the development of metal anodes with an enhanced capacity for lithium-ion batteries. Additionally, owing to its distinctive cavity structure and host–guest interaction, carbon dots can also serve as effective templates for synthesizing functional metal electrode materials. Lithium vanadate (Li_1+α_V_3_O_8_) is widely acknowledged as a promising cathode material in rechargeable lithium batteries due to its exceptional attributes, such as high capacity, excellent cycling performance, and impressive rate capability. For instance, Cheng et al. [68] reported a facile solution route and fabricated a macaroni-like Li_1.2_V_3_O_8_ cathode nanomaterial through a β-CD-based template (Figure 5a).

The hydrophilic surface of the β-CD-based template absorbed the mixture of the hydrolysate of NH_4_VO_3_ and the Li_2_CO_3_ mixtures, forming the gel precursors of Li_1.2_V_3_O_8_ after the evaporation of water, which then are calcined at 500 °C in air for removing β-CD to generate the macaroni-like nanoparticle. The as-made macaroni-like cathode delivered a highest initial capacity of 189 mAh g^−1^ at 0.1 C but an unsatisfactory cycling performance. In 2013, Chen et al. [90] fabricated stick-like CuS hierarchical structures using β-CD as the ligand and structure-directing agent by a hydrothermal approach used in LIBs. The fabricated hierarchical structures show a thickness of 25 nm stacked by tens to hundreds of the orderly self-assembled nanoplates. An investigation is conducted on the utilization of CuS as the electrode material in LIBs. The initial discharge capacity of stick-like structures in LIBs is 94 mAh g^−1^ and maintained 93 mAh g^−1^ after 30 cycles. In 2014, Wang et al. [91] conducted a study on the production of hierarchical porous carbon spheres with graphitic properties for their application in lithium-ion batteries (LIBs). In this approach, the obtained graphitic carbon spheres were prepared using α-CD, F127, and cobalt gluconate through hydrothermal synthesis followed by annealing. The cobalt gluconate accelerates the construction of the carbon structure by inducing the agglomeration of F127 micelles, leading to the precise threading of α-CD by hydrogen-bonding interactions. Another function of cobalt gluconate is to serve as a precursor for the Co catalyst, which is utilized in the catalysis of the graphitization reaction at low temperatures. These artificially produced carbon spheres with a graphitic structure exhibit an impressive reversible capacity of approximately 700 mAh g^−1^ when subjected to a current density of 50 mA g^−1^, showcasing excellent cycling stability. Notably, even under high current-density conditions (30 A g^−1^), these spheres demonstrate remarkably outstanding high-rate performance by achieving a capacity of 290 mA h g^−1^.

Because of their inherent lithium-ion properties and high theoretical specific capacity, vanadium oxides and their derivatives have emerged as extremely encouraging contenders for electrode materials in lithium batteries [92,93,94]. However, the poor electrochemical performance at a high rate and the low rate of lithium transport remain obstacles to the commercial application of vanadium oxides. Three-dimensional nanostructure materials are proven to be a great choice to suppress the above issues. Liu et al. [95] synthesized a flower-like 3D ammonium vanadate nanomaterial NH_4_V_4_O_10_ (Figure 5b) by hydrothermal reaction based on β-CD without other complex equipment. This β-cyclodextrin-based flower-like structure was proposed, providing more channels for Li^+^ and resulting in enhanced migration rate of Li^+^ owing to the obtained mesopores (the average size is 3 nm) on the surface of these flower-like structures. The reported mesoporous nanoflowers present a capacity of 242.8 mAh g^−1^ at 200 mA g^−1^ with a working voltage from 2.0 to 4.0 V. Moreover, at high current density of 1000 mA g^−1^, a capacity of 103.5 mAh g^−1^ was retained (retention 64.9%) after 200 cycles.

### 2.2. CDs in LSBs

**Figure 6:**
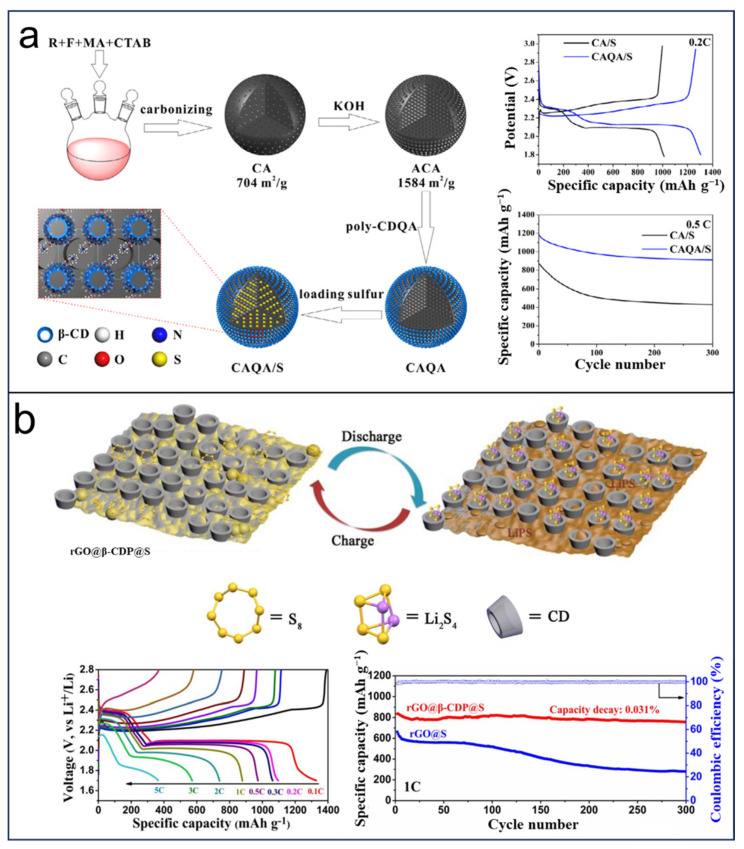
Impeding of dissolution of polysulfides by (**a**) CAQA/S and (**b**) rGO@β-CDP@S with electrochemical performance. Reprinted with permission from [96,97]. Copyright Elsevier, 2019.

LSBs have emerged as highly promising contenders for future energy storage devices, garnering considerable interest owing to the impressive theoretical specific capacity of 1675 mAh g^−1^ and an energy density of 2600 Wh kg^−1^ exhibited by sulfur cathodes [28,29,45]. In addition, the element sulfur holds the benefits of affordability, high abundance, and being environmental-friendly, while the low electrical conductivity, dissolution of polysulfides, and volume expansion of sulfur electrodes during discharge have become urgent problems, which could decrease the cycle life, specific capacity, and energy efficiency. Therefore, impeding the volume expansion of sulfur, inhibiting the dissolution of polysulfides, improving conductivity and shortening the transition length of both Li^+^ and electrons require the modification of the sulfur cathode with functional materials. In addition, CDs have shown potential application in the sulfur electrode. In 2018, Yu et al. [96] reported an electric-field-responsive surface film (CAQA/S) with a hierarchical porous structure, synthesized by blending the interconnected 3D matrix (poly-CDQA) and quaternary ammonium group (Figure 6a). The as-prepared structures exhibit the favorable features of a highly porous structure, a muti-size channel, and uniformly distributed sulfur elements. The poly-CDQA-assisted carbon material has good adsorption of sulfur and effectively switchable channels resulting in the considerably enhanced performance of LSBs. The prepared cathode delivered an initial discharge capacity of 1307 mA h g^−1^ at 0.2 C, with 84% retention after 100 cycles. The anchoring function impeded the impact of polysulfide ions on the shuttle phenomena. This new strategy provides an approach for the development of LSBs. In 2018, Ni et al. [97] constructed a ternary supramolecular nanostructure named rGO@β-CDP@S via chemical grafting of β-cyclodextrin polymers (β-CDPs) onto reduced graphene oxide nanosheets (Figure 6b), resulting in the reversible capturing and releasing of lithium polysulfides through the receptor mechanism. As shown in Figure 6b, the β-CD-modified cathode rGO@β-CDP@S exhibits a higher specific capacity of 1329 mAh g^−1^ at 0.1 C, as well as a high rate capability of 387 mAh g^−1^ at 5.0 C. Especially, the capacity decay of 0.040% and 0.053% per cycle at 2.0 and 3.0 C after 1000 cycles showed better electrochemical performances compared with the no-β-CD-modified graphene-coated composite.

**Figure 7:**
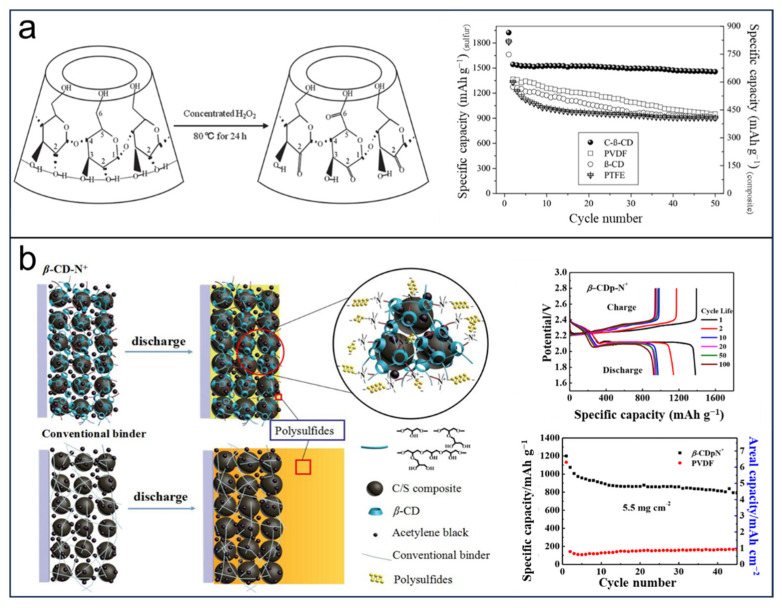
Applications of CDs-based binders in LSBs: (**a**) The formation of carbonyl-β-cyclodextrin (C-β-CD) and cycle performance. Reprinted with permission from [98]. Copyright Wiley, 2013. (**b**) Schematic representations of cathode configurations based on hyperbranched binder β-CDp-N^+^ and cycle performance. Reprinted with permission from [99]. Copyright American Chemical Society, 2015.

In the LSBs, binders play a crucial role in improving electrochemical performance, which integrates active and conductive materials together on current collectors, as well as in keeping the electrode integrated. Generally, an ideal binder should match the requirements of the constraining polysulfides, preventing the shuttle effect and improving cycling stability. In 2013, Wang et al. [98] prepared a novel binder-based modified β-CDs (Figure 7a) by partially converting the hydroxyl groups through a carbonylation process involving H_2_O_2_. The solubility of C-β-CD in water is higher than that of β-CD at room temperature (ca. 100 times), which presents an especially strong bonding capability that makes C-β-CD favorable for a binder in a battery. The as-prepared sulfur-based composite shows a sulfur utilization reaching nearly 92.2%, leading to a higher reversible capacity of 1542.7 mA h g_(sulfur)_^−1^ and excellent cycling performance. In total, 1456 mA h g_(sulfur)_^−1^ remained after 50 cycles in the discharging process. The findings indicated that the utilization of β-CD as a binder in the sulfur cathode exhibited superior performance compared to traditional binders due to the formation of C-β-CD, which effectively enveloped the surface and inhibited the clustering of the sulfur composite. These researches demonstrate that binders based on multidimensional structures with massive hydroxyl groups can ease the sulfur volume expansion in LSBs. Inspired by these ideals, Zeng et al. [99] designed a multifunctional binder (β-CDp-N^+^) by the polymerization of β-CD introduced with quaternary ammonium cation (Figure 7b). Interestingly, the enhanced mechanical stability provided by the highly branched network architecture formed in the β-CDp-N^+^ binder facilitates the effective accommodation of sulfur particles in LSBs, enabling their efficient charge and discharge with minimal volume expansion. Furthermore, the extensive presence of hydroxyl and ether groups in β-CDp-N^+^ promotes effective interactions between active and conductive substances. As a result, this cathode based on β-CDp-N^+^ exhibits an exceptional areal capacity of 4.4 mAh cm^−2^ due to these advantageous enhancements.

In 2015, Jin et al. [100] reported a novel ternary composite with a multilayer structure composed of graphene/sulfur/carbon and fabricated by a two-step synthesis based on β-CD. The β-CD played a crucial role for the generation of multilayer structures. After 200 cycles, the prepared graphene/sulfur/carbon nanocomposites maintained a reversible capacity of 800 mAh·g^−1^, 600 mAh·g^−1^ and 450 mAh·g^−1^ at 0.5 C, 1 C and 2 C, respectively. 

Although CDs have exhibited great superiority in LSBs, there was no method of evaluating the trapping behaviors and revealing the trapping mechanism of CDs toward lithium polysulfides, which are significantly important for further investigation of LSBs. In 2020, Ren et al. [101] compared the trapping behaviors of β-CD, γ-CD, and α-CD, which were used as a model platform due to their distinct and precisely defined structure and massive hydroxyl binding sites toward lithium polysulfides. The trapping performances of CDs toward Li_2_Sx species follow the order of β-CD > γ-CD > α-CD. Detailed DFT calculation demonstrates that the asymmetrical arrangement of primary hydroxyl groups play a critical role in determining trapping capacity. The result gives a deep insight for a novel structural design for electrodes in LSBs.

### 2.3. CDs in Other Batteries 

**Figure 8:**
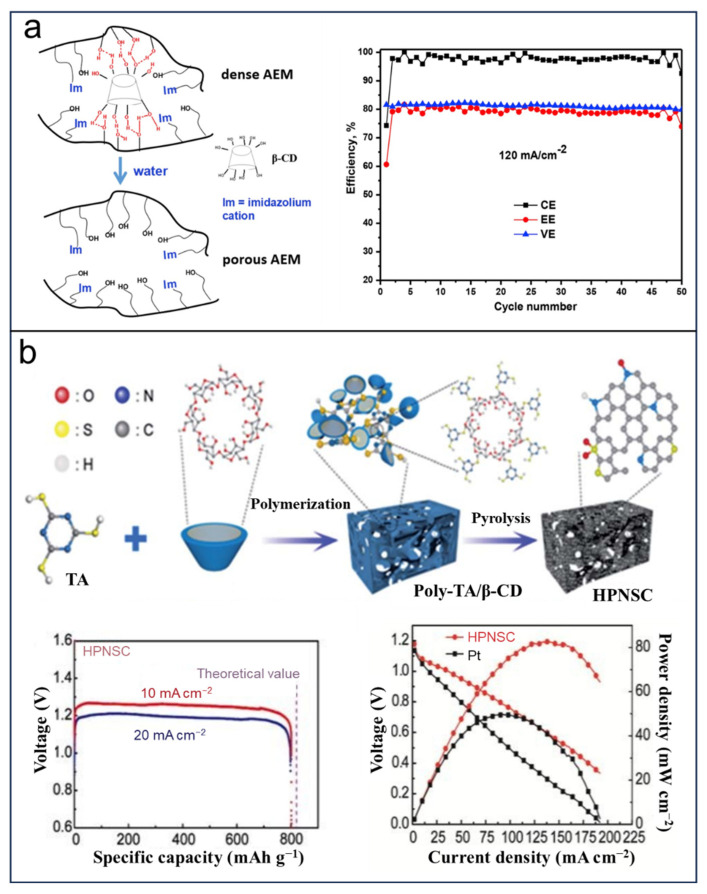
Application of CDs as templates in vanadium redox flow battery (**a**) and zinc–air batteries (**b**). Reprinted with permission from [106,108]. Copyright Elsevier, 2019 and Royal Society of Chemistry, 2019.

The vanadium redox flow battery (VFB), due to its long life, independent power, and capacity design as well environmental friendliness, has attracted much attention in EES/EEC [102,103,104]. The ion transport membrane plays a key role for suppressing the vanadium ions; permeation, resulting in better performance of the VFB [103,105]. An ideal ion transport membrane should require good conductivity and stability, which have still hindered the commercial application of VFB. Considerable attempts have been dedicated to enhancing the conductivity and stability, leading to the discovery that a substantial number of nanopores within an AEM could potentially address the aforementioned issues. CDs have proven to be great templates for the construction of function materials [89,90,91]. For instance, Ma et al. [106] fabricated a nanoporous anion exchange membrane (AEM), employing β-CD as the template (Figure 8a); because of the abundant hydroxyl groups, β-CD can disperse well in the matrix of HPSf-Im via hydrogen bonding. After the removal of β-CD, nanoporous AEM was obtained, in which the small pore structure can selectively transport protons and anions. The VFB employing such membranes demonstrated a high coulombic efficiency of 99%, and the stable energy efficiency reached 80% at 120 mA cm^−2^. In 2020, Li et al. [107] reported a strategy that uses a water-insoluble organic ferrocene as the guest and a water-soluble organic hydroxypropyl-β-CD as the host and obtained an electrochemically active electrolyte applied in aqueous organic flow batteries (AOFBs). In this approach, the mitigated degradation of ferrocenium ions leads to an improvement in cycling performance (capacity fading rate reaches 0.0073% per hour). 

Zinc–air batteries have proven to be cost-optimal to the energy conversion between the two electrical and chemical energies because of the high-capacity density of metallic zinc and the low redox potential. In 2019, Cheng et al. [108] put forward a simple approach for synthesizing HPNSC for Zn–air batteries with a new robust network based on a β-CD polymer (Figure 8b) accompanied by the generation of record-high mass activity considered a crucial factor int the practical application of Zn–air batteries in energy devices. The designed robust network serves as a self-template to form a hierarchically porous structure and electrochemically active surface area (ECSA) with abundant mesopores and macropores, which favor the mass transport. The ECSA in HPNSC reached 429 m^2^ g^−1^. The as-fabricated zinc–air battery based on HPNSC delivers a maximum power concentration of 144 mW cm^−2^ and an energy density of 1007 Wh kg_Zn_^−1^.

### 2.4. CDs in Supercapacitors

**Figure 9:**
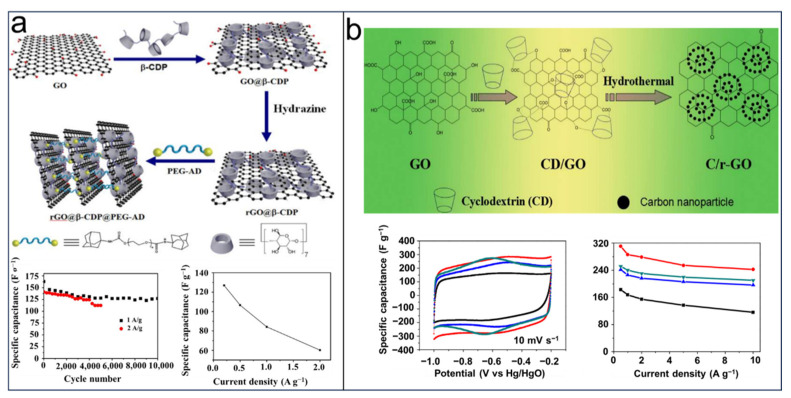
Mechanism of improving electrochemical performance of CDs modified rGO in supercapacitors: (**a**) prompting the link of rGO layers through host–guest interactions by CDs for increasing the interlayer spacing of rGO sheets (black: C/r-GO−1:1, red: C/r-GO−1:3, blue: C/r-GO−1:5, green: r-GO). Reprinted with permission from [109]. Copyright Elsevier, 2017. (**b**) Expansion of the interlayer spacing of graphene by anchoring carbonized β-cyclodextrin. Reprinted with permission from [110]. Copyright American Chemical Society, 2016.

**Figure 10:**
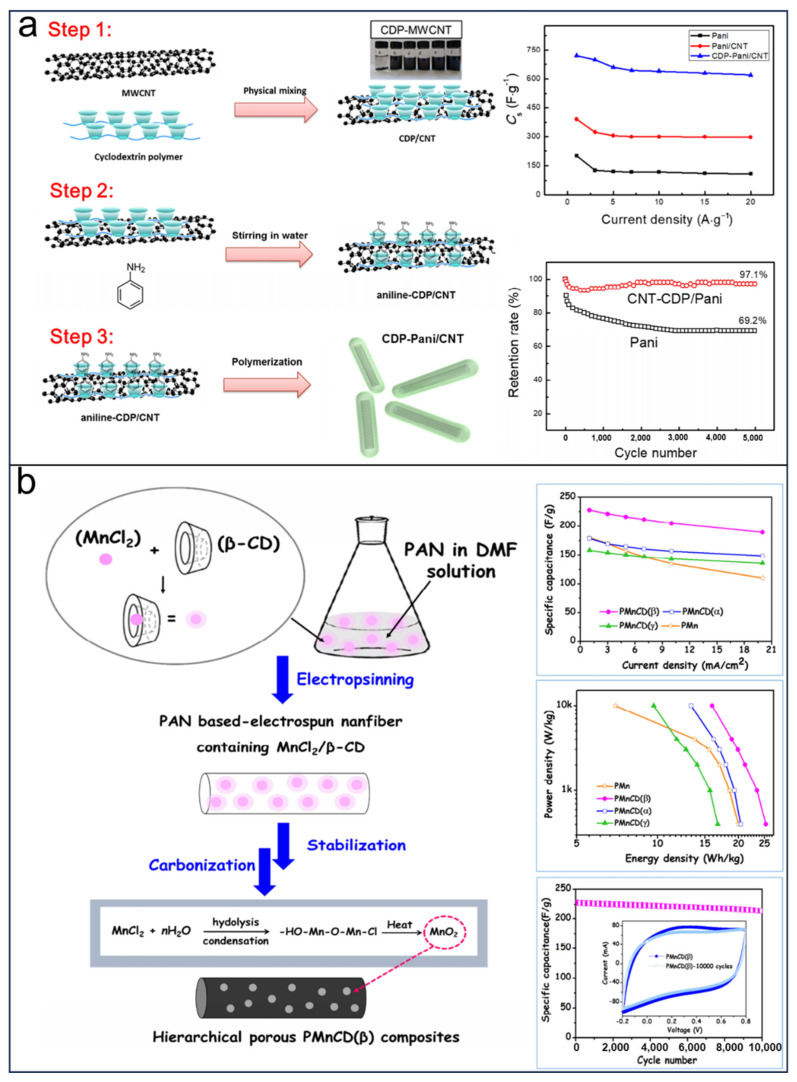
Scheme illustration of CDs used in carbon nanomaterial modifications for better electrochemical performance of supercapacitors: (**a**) anchoring aniline monomer by guest-recognition resulting in good capacitance. Reprinted with permission from [111]. Copyright Elsevier, 2020. (**b**) The fabricating process of hierarchical MnO_2_-embedded CNFs by CDs. Reprinted with permission from [112]. Copyright Elsevier, 2020.

Besides the significant applications in various batteries, CDs were extensively investigated for application in supercapacitors, which have shown great potential in EES/EEC due to the long cycling stability, excellent capability of pulsed charging and discharging, as well as their power performance in the short term. Similar to batteries, the electrochemical performance of supercapacitors is largely determined by the components of electrode-required conductivity, high capacitance, and a long cycling life [11,12,13]. In the past few years, there has been a growing interest in utilizing graphene-based materials and carbon nanomaterials as electrode materials for supercapacitors. This is primarily due to their exceptional electrical and thermal conductivity properties, which effectively meet the demands of high-energy storage. Nevertheless, the disadvantages of high resistance, self-aggregation, and poor dispersal in water still limit their applications. Modified CDs have provided a novel strategy for addressing these issues. In 2016, Ni et al. [109] produced a three-dimensional assembled graphene material (rGO@β-CDP@PEG-AD) through the utilization of host–guest interactions between β-CD polymers (β-CDP) and an end-capped poly(ethylene oxide) polymer linker (PEG-AD). The incorporation of an adamantine PEG-AD linker into rGO sheets via these interactions resulted in an expansion of the interlayer spacing between rGO sheets, facilitating efficient electron transition in three dimensions and providing pathways for ion diffusion. Figure 9a illustrates the electrode based on the fabricated rGO@β-CDP@PEG-AD, which exhibited a high initial specific capacitance of 163 F g-^1^ at a discharge current density of 1 A g^−1^. Furthermore, it demonstrated favorable cycling stability, with retention rates of 80% after 10,000 cycles at 1 A g^−1^ and 82% after 5000 cycles at 2 A g^−1^. Another team, led by Huang [110], successfully produced graphene-based materials through the application of hydrothermal reduction and carbonization techniques on a supramolecular hydrogel that was prepared by connecting β-CD to graphene oxide. The interlayer spacing between the graphene nanosheets was expanded by incorporating carbonized β-CD, which effectively prevented aggregation and restacking of the graphene. This modification greatly enhanced the ion and electron transport. The resulting carbon nanoparticles exhibited remarkable electrochemical performance, with a specific capacitance of 310.8 F g^−1^ at 0.5 A g^−1^; exceptional rate capability (242.5 F g^−1^ at 10 A g^−1^); and excellent cycle stability even after undergoing 10,000 cycles at a scan rate of 50 mV s^−1^ (Figure 9b). Zhang et al. [111] developed a method of utilizing β-CD to create CD polymer (CDP) functionalized polyaniline/carbon nanotubes, taking advantage of the stable dispersion in water and the strong recognition ability of β-CDp towards aniline monomer. The resulting composites (Figure 10a) successfully combined the excellent electrical conductivity of CNTs with the favorable capacitance properties of Pani. When used as electrodes in a symmetric supercapacitor device, the CDP-Pani/CNT electrodes exhibited a specific capacitance of 107.4 F·g^−1^ at 1 A·g^−1^, which remained above 97% even after undergoing 5000 cycles at 5 A·g^−1^. In another study conducted by Jeong et al. [112] in 2020, they introduced hierarchical porous carbon nanofiber (CNF)/MnO_2_ composites (PMnCD) based on polyacrylonitrile (PAN)/CD materials (Figure 10b). The PMnCD(β) electrode possesses a hierarchical porous structure with a larger specific surface area, allowing for the fast diffusion of electrolyte ions and enhanced attractive interactions. Additionally, the electrode exhibits a specific capacitance of 228 F g^−1^ at 1 mA cm^−2^, achieves a maximum energy density ranging from 25.3 to 16.0 Wh kg^−1^ at power densities between 400 and 10,000 Wk g^−1^, and demonstrates excellent cycling stability in an aqueous solution, with only a slight decrease of 6% after undergoing 10,000 cycles.

Here, we summarize the electrochemical performance of EES/EEC devices based on CDs in Table 1. Noticeably, CDs played a key role in increasing the electrochemical performance of batteries and supercapacitors, but it is still very painful to achieve the practical application of CDs in EES/EEC. More subtle molecule design strategies are required to obtain higher stability, a specific capacity or capacitance, the cycle life and low cost. Future research on CDs may give more attention to COFs and couple redox-active sites with CDs, making CDs participate in the redox reaction to overcome the negative impacts when used as additives.

## 3. CAs in Electrochemical Energy Storage and Conversion

CAs are the outcomes resulting from the condensation process of p-substituted phenols and formaldehyde via base induction firstly obtained by Zinke et al. in 1942 but named by C. D. Gutsche because of the structure resembling a Greek vase [61]. There are inherent macrocyclic cores in the molecular structures with tunable sizes which favor the host–guest interaction, and the methylene group attached to the benzene ring make CAs selectively functionalized. Due to these distinct features, CAs have been synthesized to many derivatives and widely used in various fields, such as ion extraction, gas sensor, catalysts, and biomedicine [113,114,115,116]. One of the most well-known derivatives is calix[*n*]quinone, which prefers redox-active sites make these macrocycles promising candidates for organic electrode material. Furthermore, the emerged alternative calixarenes have been used in EES/EEC for better electrochemical performance.

### 3.1. CAs in LIBs

**Figure 11:**
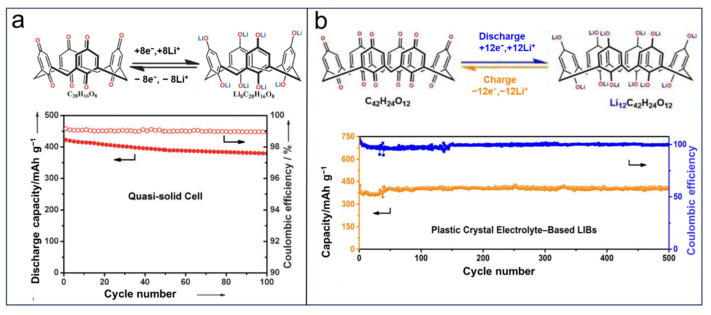
(**a**) Proposed electrochemical redox reactions of C4Q to give Li_8_C_28_H_16_O_8_ and cycle performance of the quasi-solid-state cells based on C4Q. Reprinted with permission from [69]. Copyright Wiley, 2013. (**b**) The mechanism of C6Q discharge–charge and cycle performance of C6Q-based cell [117]. Reprinted with permission from [116]. Copyright Elsevier, 2020.

LIBs are still limited by low safety and environmental friendliness for further commercial applications [3,5]. It has been proven that organic electrode materials could be promising candidates for the next generation of friendly batteries owing to the benefits provided by their high theoretical specific capacity, strong designability of molecular structures, good reversibility, high security, and low cost. Calix[*n*]quinones (CQs) have received much attention because of their redox-active sites. Huang et al. [69] were the first to introduce calix[4]quinone (C4Q) as a cathode material in LIBs, demonstrating a theoretical capacity of 446 mAh g^−1^ (Figure 11a). In this study, an electrolyte consisting of LiClO4 dissolved in DMSO and loaded with PMA/PEG-based GPE was employed. As shown in Figure 11a, the initial discharge capacity of the quasi-solid-state cells prepared was found to be 422 mAhg^−1^, accompanied by an average potential of 2.64 V. Furthermore, after undergoing charge–discharge cycles at a rate of 0.2 C for a total of 100 cycles, the remaining capacity was determined to be 379 mAh g^−1^. However, the batteries based on C4Q were hindered by the poor cycling stability (capacity approximating to 100 mAh g^−1^ after five cycles at high rate.) owing to the solubility of organic electrolytes and interfacial resistance of the quinone cathode. To address these issues, the same research group synthesized calix[6]quinone (C6Q) composed of six p-quinones linked via methylene groups at the ortho-positions, resulting in 12 redox-active sites of C6Q for the lithiation reaction (Figure 11b) [117]. The lower solubility of C6Q makes it a suitable choice for use as an active electrode material. Consequently, the optimized battery utilizing a plastic crystal electrolyte (PCE) demonstrates a capacity maintenance of 405 mAh g^−1^ after 500 cycles. Additionally, C6Q possesses the same theoretical specific capacity (Ctheo = 446 mA h g^−1^) but has a higher molecular weight (MC4Q = 480, MC6Q = 720). Notably, C6Q features a larger cavity that allows for increased penetration of electrolytes, which is advantageous for lithiation/delithiation processes. In a study conducted by Huang et al. [118] in 2020, C6Q was synthesized and applied in LIBs, exhibiting an impressive initial capacity of 423 mA h g^−1^ at 0.1 C in LIBs and maintaining capacities of 216 and 195 mA h g^−1^ after undergoing100 and 300 cycles, respectively.

**Figure 12:**
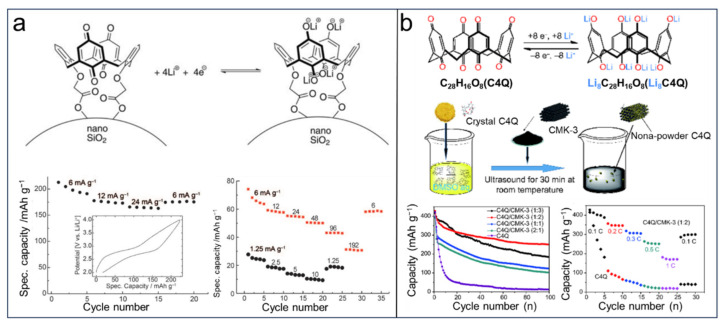
(**a**) Calix[4]arene grafted on a silica nanoparticle and the proposed redox reaction (top) and cycling performance of CQ grafted on carbon black and SiO_2_ (bottom). Reprinted with permission from [119]. Copyright Wiley, 2010. (**b**) Redox insertion/extraction mechanism of C4Q and the preparation of the composites, and the cycling performance of the composites. Reprinted with permission from [120]. Copyright Springer, 2018.

To improve the conductivity and insolubility of organic cathode materials, using composites is an alternative method. For instance, Genorio et al. [119] proposed the concept of grafting calix[4]arene (C4Q) onto the surface of solid nanoparticles (silica nanoparticles and carbon black), which has the potential to enhance the durability of organic molecules during the electrochemical process and allows changes in its electrochemical properties. The results showed that the cycling stability is obviously improved compared with nongrafted materials, but it inevitably loses part of the capacity (Figure 12a). Zheng et al. [120] reported the organic composites electrode materials by combining C4Q and mesoporous carbon (CMK-3) (Figure 12b). The as-made composites integrate the high energy density of C4Q and superior conductivity of CMK-3, which relieved the dissolution issue and optimized the electroconductivity. As shown in Figure 12b, the composite (1:2) exhibits an excellent initial capacity of 427 mA h g^−1^ for LIBs; however, its capacity retention rate drops to only 58.7% after undergoing 100 cycles.

Electrolytes, another key component of batteries, play a crucial role in elevating the electrochemical performance of LIBs. An ideal electrolyte in LIBs is expected to have great conductivity of lithium cation. The special cavity with adjustable sizes makes CAs promising additives to electrolytes. For example, Scrosati et al. [121] designed a new type of composite polymer electrolyte using two calix[4]urea derivatives as an anion-trapping additive. The consistent enhancement of the lithium transference number demonstrated that the calixarene additive can trap lithium ion. The prepared electrolytes showed a lithium-ion transport number equal to 1. Calix[6]pyrrole derivatives (C6P) are selective on larger anions (I^−^, CF_3_SO_3_^−^, BF_4_^−^). Kalita et al. [122] applied C6P in poly(ethylene oxide) (PEO)- based electrolytes, significantly changing the lithium transference number to values close to 1. These studies demonstrated that the CAs can capture the lithium cation, leading to the enhancement of lithium cation conductivity, which provides guidance for the further study of electrolytes.

### 3.2. CAs in SIBs

**Figure 13:**
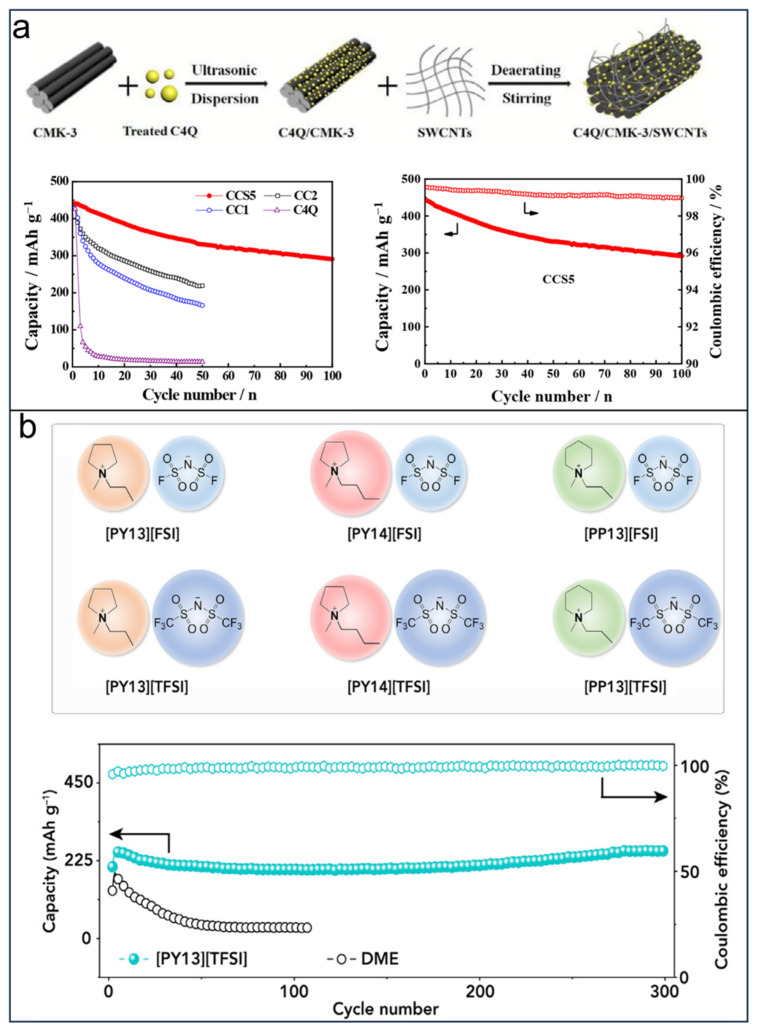
(**a**) Preparation process and three-dimensional model of C4Q/CMK-3/SWCNTs nanocomposites and cycle performance of C4Q/CMK-3/SWCNTs. Reprinted with permission from [124]. Copyright Royal Society of Chemistry, 2019. (**b**) Chemical structures of six ionic liquids and cycling stability of C4Q cathode. Reprinted with permission from [126]. Copyright Elsevier, 2019.

SIBs have the potential to serve as a viable option for the future generation of rechargeable batteries, similar to LIBs. The sodium element, the same main group element with lithium, shows similar alkali metal chemistries with Li, as well as earth-abundant resources [8,30]. Compared with metal oxide cathode materials, organic-based electrode materials have attracted much attention due to the virtue of high capacity, feasible structure designability, and infinite availability [41]. Calix[*n*]quinone, a type of derivative of calix[*n*]arene, has been used for SIBs and has demonstrated impressive electrochemical performance. However, the cycle stability and practical energy of quinone-based SIBs are still limited due to the solubility of quinone in electrolytes that are based on organic solvents. To address these issues, many efforts have focused on composites and electrolytes. Composites have proven to be an effective strategy to overcome the solubility issue of calix[*n*]quinone by grafting CQs onto conductive and stable platforms. For example, inspired by the application of calix[4]quinone (C4Q) in LIBs, which gained an initial capacity of 422 mAh g^−1^, Zheng et al. [123] employed C4Q-based composites as the cathode materials for rechargeable sodium batteries. The optimized nanocomposite demonstrated an excellent initial discharge capacity of 438 mAh g^−1^ at 0.1 C and maintained a capacity of 219 mAh g^−1^ after undergoing 50 cycles due to the advantageous effects of nanoscale dimensions and efficient conduction provided by mesoporous carbon. This approach presents a viable strategy for enhancing the conductivity of C4Q by integrating it with highly conductive and stable nanomaterials. In 2019, Yan et al. [124] introduced single-wall carbon nanotubes (SWCNTs) into the C4Q/CMK-3 system and formed C4Q/CMK-3/SWCNTs composites with a three-dimensional network, as shown in Figure 13a. The composites of C4Q/CMK-3/SWCNTs (40 wt%, 40 wt%, 10 wt%) utilized as cathodes for SIBs exhibit a remarkable reversible capacity of 290 mAh g^−1^ after undergoing 100 cycles at a rate of 0.1 C, showcasing superior cycling performance in comparison to the C4Q/CMK-3 composites (Figure 13a). In addition, the combination of C4Q and biocarbon has also been reported for economic reasons [125]. Another strategy is to use suitable electrolytes to prevent the dissolution of quinone. Wang et al. [126] conducted a study on the use of ionic liquid (IL) as electrolytes, specifically *N*-methyl-*N*-propylpyrrolidinium bis(trifluoromethanesulfonyl)amide ([PY13][TFSI]). These IL-based electrolytes were found to have an inhibitory effect on the dissolution of C4Q due to their weak donor ability and high polarity. The utilization of the [PY13][TFSI]-based electrolyte resulted in a C4Q cathode with exceptional cycling stability, maintaining 99.7% retention at 130 mA g^−1^ for 300 cycles (as shown in Figure 13b). This research successfully demonstrated the potential of employing IL-based electrolytes as a strategy to enhance the electrochemical performance of SIBs, leading to significantly improved energy density reaching up to 863 Wh kg^−1^.

### 3.3. CAs in Other Batteries

**Figure 14:**
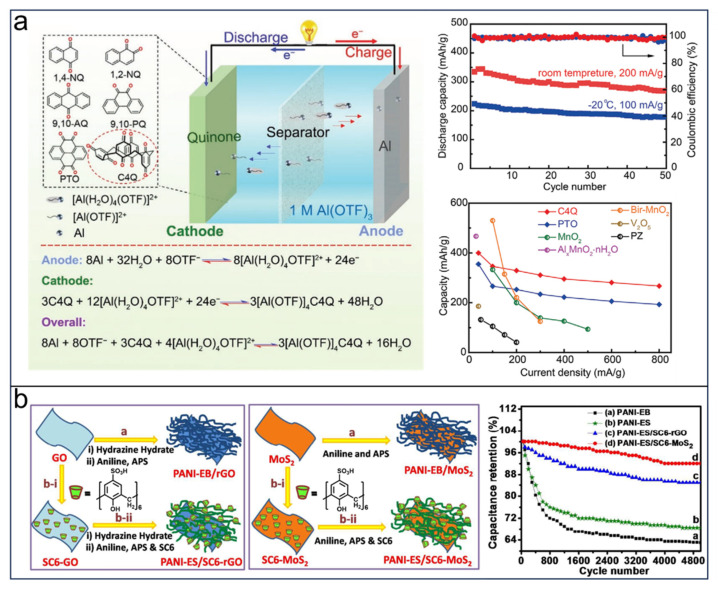
(**a**) Schematic illustration of fabricated aqueous Al-quinone batteries and cycling performance, and rate performance of C4Q cathode. Reprinted with permission from [129]. Copyright Wiley, 2021. (**b**) Schematic diagram of the procedure for preparing PANI-ES/SC6-rGO, PANI-ES/SC6-MoS_2_ nanocomposite, and cycle performance. Reprinted with permission from [132]. Copyright Royal Society of Chemistry, 2017.

Rechargeable aqueous aluminum batteries (AABs) are expected to be potential alternatives for EES/EEC because of the advantages of high specific capacity, low cost, and air/water insensitivity [127]. The trivalent Al^3+^ can lead to a strong Coulombic interaction among cathode materials and anions in electrolytes that decrease the kinetics of Al^3+^ diffusion and collapse the host structure, thus leading to high overpotential, low cycling performance, and low rate capacity [128]. Therefore, the storage of Al^3+^ becomes a key factor in the improvement of the performance of AABs. Based on the ion coordination mechanism of quinone, C4Q has been applied as cathode materials for AABs. Li et al. [129] investigated the utilization of C4Q, which consists of four multi-carbonyls–quinone unit, as the cathode material for AABs. The anode employed was Al, and the electrolyte used was a 1.0 M aqueous solution of aluminum trifluoromethanesulfonate (Al(OTF)3). Both experimental and computational analyses revealed that carbonyl groups have the ability to interact with [Al(OTF)]^2+^ ions during discharge. As illustrated in Figure 14a, the AABs incorporating the prepared C4Q cathode demonstrated remarkable performance characteristics: the highest capacity (400 mAh g^−1^), the lowest polarization (42 mV), and excellent cycling stability (81% capacity retention after 50 cycles). Furthermore, these AABs also exhibited outstanding performance at low temperatures (−20 °C), achieving a capacity of 224 mAh g^−1^.

Other aqueous batteries, zinc batteries (ZBs), have shown promising application for future large-scale energy storage and conversion because of the advantages of high-capacity density (~5855 mA h cm^−3^), low redox potential, large production, and good compatibility with water [130]. Cathode materials play an important role in the performance of ZBs’ required large reversible charge/discharge capacities and long cycle life. Inspired by the literature, quinone-based materials in ZBs as cathode materials have also been investigated. In 2018, Zhao et al. [131] utilized a high-performance cathode in ZBs along with a membrane that selectively allows cations to pass through. When used in an aqueous electrolyte, the cathode based on C4Q exhibited a remarkable capacity of 335 mA h g^−1^ by utilizing six carbonyls, maintained a stable operating voltage of 1.0 V, showed minimal polarization (70 mV), and achieved an impressive energy efficiency of 93% at low current density. Notably, it demonstrated exceptional durability, with 87% capacity retention after undergoing 1000 cycles at 500 mA g^−1^.

### 3.4. CAs in Supercapacitors

Due to the cavity structure and template function, CAs have proven to be of significance to EES/EEC devices, as well as supercapacitors. In 2017, Waghmode et al. [132] synthesized a functionalized 4-sulphatocalix[6]arene hydrate (SC6) used as a stabilizer for supercapacitors. After the addition of SC6, stabilized composites termed as PANI-ES/SC6-rGO and PANI-ES/SC6-MoS_2_ containing 2D rGO/MoS_2_ nanosheets were obtained, in which SC6 increases the surface area of 2D nanosheets and simultaneously keeps the electrical conductivity. Significantly, SC6 offers the advantage of preventing the re-stacking of rGO/MoS_2_ nanosheets by attaching PANI-ES nanostructures onto the surfaces of rGO/MoS_2_. Consequently, the PANI-ES/SC6-MoS_2_ composites produced demonstrate an exceptional specific capacitance of 691 F/g at 1 A/g while exhibiting remarkable durability (retaining 91% of specific capacitances after 5000 cycles, as shown in Figure 14b).

At the end of this section, we compare the electrochemical performance of EES/EEC devices based on CAs in Table 2. The current applications of CAs are mainly focused on the redox-active carbonyl groups, which are favorable for increasing the specific capacity. The results demonstrated that with more carbonyl groups, a higher special capacity can be obtained because of a higher carrier coupling ability. Therefore, the molecular design strategies of CAs in EES/EEC may put the attention on synthesizing larger macrocyclic molecules with more redox-active sites.

## 4. CBs in Electrochemical Energy Storage and Conversion

Cucurbit[*n*]urils (CB[*n*]) are formed by the condensation reaction between glycoluril and an abundant amount of formaldehyde, typically consisting of five, six, seven, eight, or ten glycoluril units [133]. Cucurbiturils were first synthesized by Behrend et al. with an unknown structure in 1905 and were disclosed by Mock and co-workers until 1981 and then named “Cucurbituril” [134]. The pumpkin-shaped cucurbiturils have a cavity with two identical openings, which distinguish them from α-cyclodextrin (α-CD), as well as a rigid structure [135]. These features also make cucurbiturils attractive for the construction of supramolecular architectures and for being extensively investigated as molecular hosts. Furthermore, it has been proven that carbonyl groups in CB[*n*] molecules can bind to alkali and alkaline earth metal ions [136]. In the family of CBs, a specific composition consisting of six glycoluril units has garnered significant interest due to its exceptional structural stability and the presence of a supramolecular framework formed through hydrogen bonding. This composition is commonly referred to as cucurbit[6]uril (CB[6]), and CB[6] being applied in EES/EEC devices as a key component has been reported in recent years.

### CBs in LSBs

**Figure 15:**
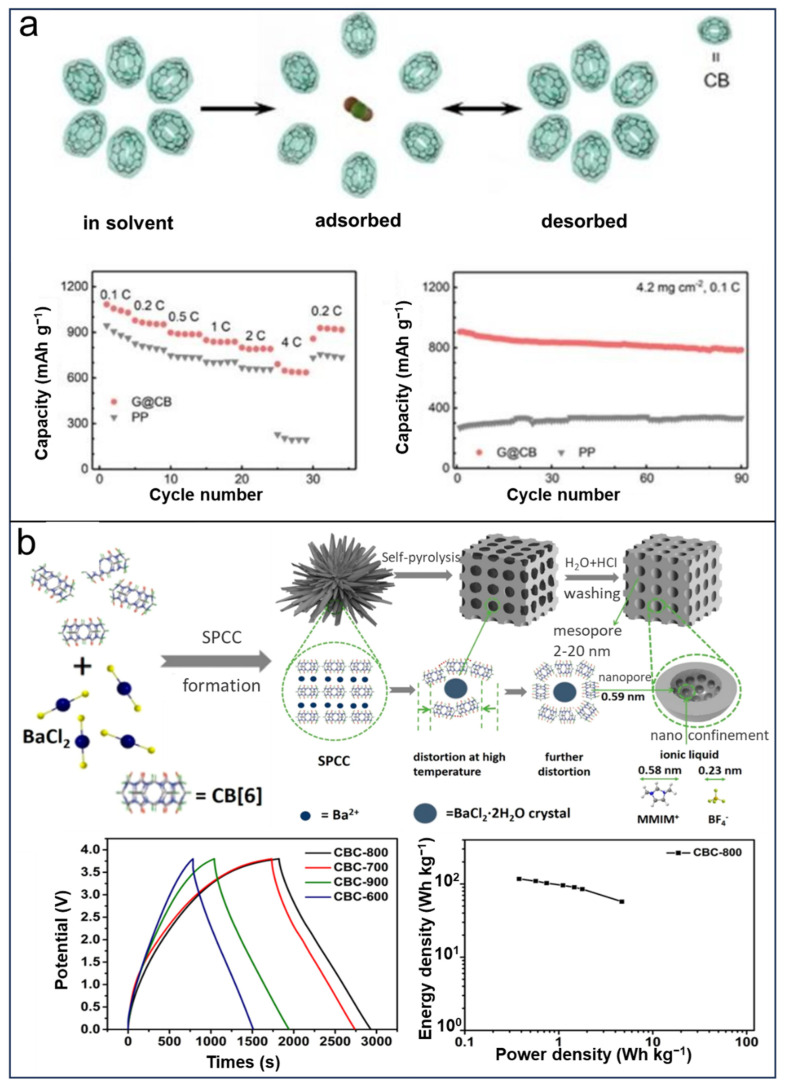
(**a**) Schematics for the “LiPS-breathing” model and cycle performance of as-fabricated LSBs based on CB[6]. Reprinted with permission from [67]. Copyright Wiley, 2017. (**b**) Formation and the following self-pyrolysis of CBC, galvanostatic charge−discharge curves, and energy density diagram of CBC electrode. Reprinted with permission from [137]. Copyright American Chemical Society, 2021.

Xie et al. [67] fabricated a separator based on CB[6] that serves as a receptor for the reversible storage/delivery of intractable polysulfides in LSBs. The use of CB[6] can control the undesirable polysulfide shuttle and greatly improve the electrochemical performance of LSBs. The capacity of as-prepared LSBs largely increased from 300 to 900 mAh g^−1^ with a high sulfur loading (4.2 mg cm^−2^) (Figure 15a). This work demonstrated that CB[6] could be a promising material used in EES/EEC due to its distinct advantages. In 2021, Cui et al. [137] reported a subnanoporous carbon (CBC) electrode based on CB[6] used in a supercapacitor. As shown in Figure 15b, the energy density of CBC electrodes reaches 117.1 Wh kg^−1^, and they demonstrate a potential window exceeding 3.8 V when immersed in an MMIMBF_4_ electrolyte within a two-electrode system. In spite of these successful application of CBs, there are still only a few studies on the application of CB[*n*] in EES/EEC. It is believed that CB[*n*] could be extensively explored for wide application in diverse batteries and supercapacitors with further research and the insights of material science.

Also, the utilization of the cavity and increasing active sites in PAs should receive more attention in the molecular design of CBs.

## 5. PAs in Electrochemical Energy Storage and Conversion

Compared to CDs, CAs, and CBs, PAs are a recently developed class of macrocycles composed of hydroquinone units connected by methylene groups at the para positions. This nomenclature was introduced by Ogoshi in 2008 [65], containing pillar-shaped redox-active structures and electron-rich cavities, and the same as cyclodextrins, PAs can easily be functionalized with bulk groups, resulting in rigid chemical structures and selective binding to guest molecules. Furthermore, compared with cyclodextrin and calixarene, PAs are highly symmetrical and rigid, affording their selective binding to guests [65,138,139]. Recently, there has been a growing interest in electrochemical energy storage and conversion using PAs, particularly those based on quinone. These PAs have garnered attention for their impressive capacity, which can be attributed to their unique pillar-shaped cavity and redox-active structures.

### 5.1. PAs in LIBs

**Figure 16:**
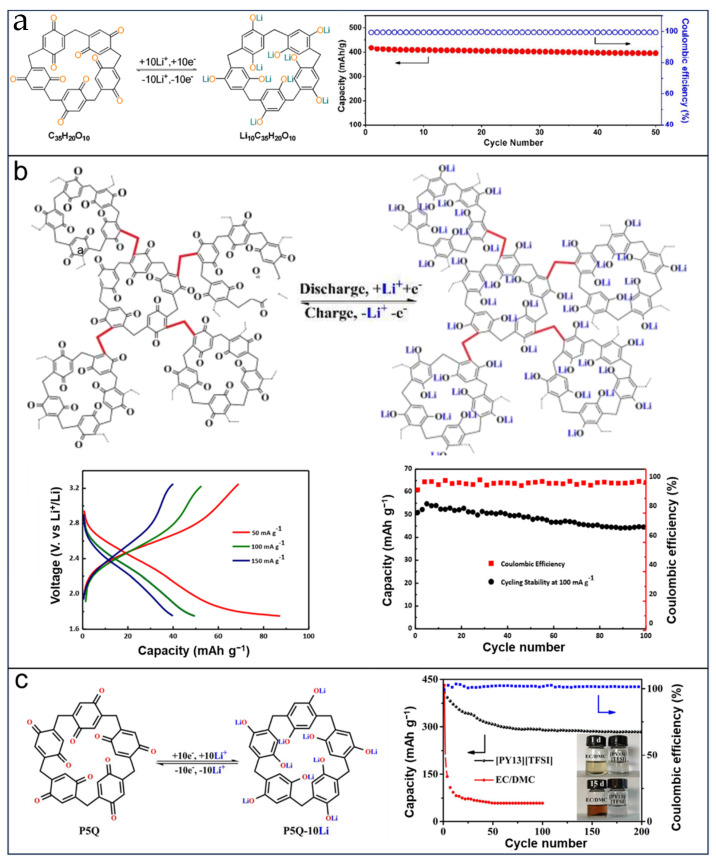
Application of pillar[5]quinone in lithium batteries: (**a**) structure and proposed electrochemical redox mechanism of pillar[5]quinone and cycling performance of the all-solid-state cell with P5Q cathode. Reprinted with permission from [140]. Copyright American Chemical Society, 2014. (**b**) Chemical structure of synthesized PolyP5Q, galvanostatic charge–discharge profile, and cycling performance of PolyP5Q. Reprinted with permission from [70]. Copyright Elsevier, 2017. (**c**) Redox diagram of P5Q and cyclic performance of P5Q-[PY13][TFSI]. Reprinted with permission from [141]. Copyright Elsevier, 2020.

Zhu et al. [140] developed a solid-state lithium organic battery using pillar[5]quinone (P5Q) as the active material and PMA/PEG-LiClO_4_-3 wt% SiO_2_ as the composite polymer electrolyte (CPE). The battery based on P5Q exhibited an average operating voltage of 2.6 V and an initial capacity of 418 mAh g^−1^ at a rate of 0.2 C, with a retention rate of 94.7% after undergoing 50 cycles (as shown in Figure 16a). These results demonstrated that P5Q can favor Li uptake as expected, but the dissolution in battery solvents remains an obstacle to such organic electrode materials for the preparation of higher performance organic batteries. Porous organic polymers (POPs) have received considerable interest from chemists due to their selectivity, stable physicochemical properties, and structural abundances. Furthermore, a porous structure can contribute to the infiltration of electrolyte ions and render the volume expansion of electrode materials, which are expected to be favorable for better electrochemical performance. Ahmad et al. [70] fabricated a novel hyper-crosslinked polymer based on poly-pillar[5]quinone (Poly-P5Q) as the electrode material in LIBs. The initial discharge capacity of the Poly-P5Q cathode was 105 mAh g^−1^, and it maintained 82.3% retention after undergoing 100 cycles at a rate of 100 mA g^−1^ within a voltage range of 1.75 to 3.25 V (as depicted in Figure 16b). In spite of the disappointing performance of porous organic polymers, the dissolution of P5Q in battery solvents still needs to be addressed. In 2020, Sun et al. [141] introduced ion liquid electrolytes into LIBs with P5Q-based electrodes. The introduction of ion liquid electrolytes makes the issues of reducing the energy density of batteries, decreased ionic conductivities, and interface resistances produced by the methods of immobilization and solid/quasi-solid electrolytes easily avoided. The as-built LIBs system with a P5Q-based cathode and 0.1 M LiPF_6_ (lithium hexafluorophosphate)/[PY13][TFSI]) electrolyte exhibits an obviously increased initial capacity reaching 408 mAh g^−1^, retaining 70% after 200 cycles at 0.2 C (Figure 16c).

### 5.2. PAs in SIBs

Because of the cavity structure, pillar[5]quinone has been applied in SIBs, which attracted much attention owing to their abundance and low cost, in which the dissolution of organic cathode materials still exists. To tackle this concern, Xiong et al. [142] devised a cathode using P5Q as the foundation, wherein they merged SWCNTs and enclosed CMK-3 with P5Q. Due to their distinctive carbonyl structure, the SIBs produced exhibited an initial capacity of up to 418 mAh g^−1^, which is close to the theoretical capacity (446 mAh g^−1^). Even after 300 cycles at 0.1 C, a capacity of 290 mAh g^−1^ was maintained. These findings suggest that pillar[*n*]quinones have potential as cathode materials in SIBs; however, their practical application may be limited by their theoretical capacity.

### 5.3. PAs in Supercapacitors

**Figure 17:**
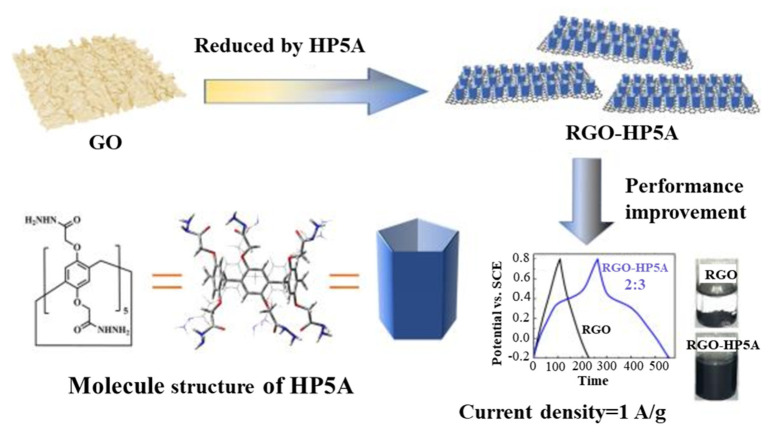
Schematic illustration of formation of RGO-HP5A and electrochemical performance of RGO-HP5A. Reprinted with permission from [143]. Copyright Elsevier, 2020.

The above approaches also can provide significant insights into the future research of LIBs and SIBs. Besides, pillar[5]quinone can also be used in supercapacitors owing to their high specific capacity and cage structure. Guo et al. [143] fabricated a composite (RGO-HP5A)-based pillar[5]arene containing reduced graphene oxide (RGO) by redox reaction (Figure 17). The water dispersion and stability of the few-layer RGO-HP5A hybrids were observed to be favorable. The nanocomposite fabricated for supercapacitors exhibited a remarkable specific capacitance of 331 F·g^−1^ at 0.5 A·g^−1^, along with an exceptional cycling performance attributed to the synergistic effect of RGO and HP5A within this composite.

### 5.4. Computation Studies of PAs in Electrochemical Energy Storage and Conversion

In spite of these experimental successes, computation studies have proven to be a powerful approach to the fundamental understanding and practical design of battery materials [144,145]. Many electrochemical properties of battery materials are determined by the structures of these materials, such as ionic conductivity, cycling life, and chemical stability. These computation approaches have been employed to screen the desirable properties of star battery materials [146]. For example, pilli[*n*]quinone has been a star battery material owing to its high capacity and cavity structure and wide investigation in LIBs and SIBs. Using density functional theory (DFT), Huan et al. [147] conducted calculations on pilli[4]-quinone’s molecular geometry, electronic structure, solvation effects, and redox potential in LIBs. Xie et al. [148] introduced two heteroatom-linked variants of pillar[4]-quinone—oxa-pillar[4]-quinone and thia-pillar[4]-quinone—exhibiting theoretical specific capacities of 659 mA h g^−1^ and 582 mA h g^−1^, respectively, when evaluated through DFT at the M06-2X/6-31G(d,p) level of theory. These oxa-pillar-[4]-quione and thia-pillar-[4]-quione compounds notably surpass conventional pillar[4]-quinone as cathode materials for LIBs in terms of their specific capacity. These findings offer novel insights that could potentially enhance the electrochemical performance of quinone-based cathode materials utilized in LIBs (Table 3).

## 6. Other Macrocycles in Electrochemical Energy Storage and Conversion

In recent years, numerous newly discovered macrocycles have garnered significant interest owing to their remarkable structural characteristics and chemical capabilities [149,150]. The existing literature has proven that many of these macrocycles have promising applications for use as electrode materials in EES/EEC [151,152,153,154,155]. In this review, we list a few representative novel macrocycles which are applied as electrode materials in LIBs, SIBs, AIBs, and ZIBs.

**Figure 18:**
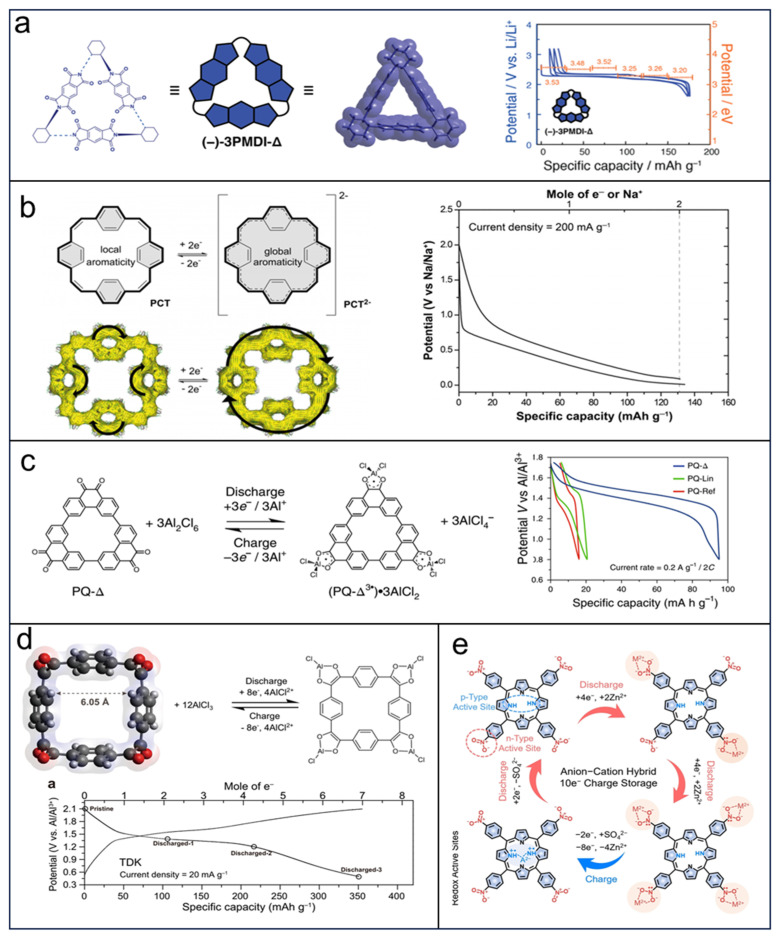
Applications of novel macrocycles in LIBs, SIBs, AIBs, and ZIBs: (**a**) The macrocyclic molecular structure of triangular PMDI-Δ and specific capacity diagram in LIBs. Reprinted with permission from [151]. Copyright American Chemical Society, 2017. (**b**) Illustration of aromatic conversion concept by PCT and specific capacity diagram in SIBs. Reprinted with permission from [153]. Copyright Wiley, 2020. (**c**) The chemical redox reaction of PQ-Δ in AIBs and specific capacity diagram. Reprinted with permission from [152]. Copyright Nature, 2018. (**d**) Illustration of discharge and charge processes of macrocycle TDK coupling with AlCl^2+^ and specific capacity diagram in AIBs. Reprinted with permission from [154]. Copyright Nature, 2021. (**e**) 10e- storage mechanism of macrocyclic TNP in ZIBs. Reprinted with permission from [155]. Copyright Wiley, 2024.

The attractiveness of rechargeable organic batteries based on redox-active organic materials lies in their low cost, high specific capacities, and environmentally friendly nature. It is worth noting that macrocycles have demonstrated superior electrochemical performance when compared to their linear counterparts. For instance, Kim et al. [151] synthesized a novel triangular macrocycle composed of three pyromellitic diimide molecules, named PMDI-Δ, and the structure is shown in Figure 18a. Then the electrochemical performances of the macrocycle in LIBs were determined through fabricated coin-type cells. The results exhibit that this PMDI-Δ has a single output voltage of 2.33 V and a specific capacity of 163 mAh g^−1^. Compared with this PMDI-Δ, the corresponding dimmer cyclic derivative or single diimide shows poor electrochemical performance. The great single voltage may be attributed to the larger space in the triangular PMDI-Δ where the electron repulsion among the reduced diimide units can be diminished in comparison with the dimmer cyclic derivative or single diimide, resulting in a more stable state for an electron. In addition, PMDI-Δ has a better cycle life compared with a dimmer cycle and single unit, owing to the poor solubility of PMDI-Δ, but the cycle life of PMDI-Δ is still unsatisfied (50 cycles).

Paradigmatically, SIBs exhibit great potential as a viable alternative to LIBs, owing to their substantial reserves. The electrochemical efficacy of the complete cell is predominantly influenced by the constituent electrode materials. Unfortunately, the anode materials commercially used in LIBs exhibit inactive features for applications in SIBs. Therefore, exploration of excellent anode materials to facilitate the development of high-performance SIBs is very significant. Additionally, macrocycles have shown many advantages when applied as electrode materials. In 2020, Eder et al. [153] synthesized a novel macrocycle (named PCT), which is composed of four benzene molecules linked by four vinylene groups through a Wittig reaction, and the authors proposed a new concept that PCT in a locally aromatic neutral state can switch to a globally aromatic state when doubly reduced (Figure 18b). This distinctive feature makes the macrocycle highly stable when used as an electrode material. The single crystal analysis demonstrates that solid PCT has large voids and the distance between neighbor PCT molecules is larger than 3.7 Å, which is large enough for sodium insertion. All these prominent features result in PCT delivering a specific capacity of 133 mAh g^−1^ at 200 mA g^−1^ and a stable cycling performance (over 500 cycles without capacity fading), with a great cycle life but unexceptional specific capacity.

Another candidate, AIBs, has a theoretical capacity of 2980 mAh g^−1^ and a third reserve, which make these batteries considerably attractive, while the multi-valency of aluminum ion causes AIBs to be hindered in fundamental researches because of the strong Coulombic interaction. In 2018, Kim et al. [152] discovered a macrocycle with a triangular structure (PQ-Δ) derived from phenanthrenequinone (PQ), which contains six carbonyl groups capable of undergoing redox reactions. Experimental findings revealed that this macrocycle exhibited a specific capacity of 94 mAh g^−1^ at 0.2 A g^−1^ when utilized as the cathode material in advanced lithium batteries (ALBs). These results further confirmed the formation of the cationic complex PQ-Δ3^•−^, which facilitated the reversible insertion and extraction of cationic chloroaluminate species (Figure 18c), and this phenomenon is also supported by the TEM and PXRD diffraction results. Furthermore, the PQ-Δ based cathode showed excellent cycle performance (maintained 82 mAh g^−1^ after 200 cycles) because of the triangular constitution restricting the solvation of PQ-Δ molecules. Then the same group reported a novel macrocycle named tetradiketone (TDK) in 2021 [154], which was synthesized from the derivatives of cyclobenzoin developed by the Miljanić and Bunz groups. There are eight carbonyl groups in the TDK macrocycle (Figure 18d), which can reversibly bond with AlCl^2+^, resulting in an excellent specific capacity of 350 mAh g^−1^ of TDK in ALBs. Another advantage of the TDK macrocycle is the large void with a diameter of 6.5 Å, which could facilitate the migration of a charge carrier. To overcome the dissolution issue, TDK macrocycles were blended into activated carbon, accompanied by a significant decrease in specific capacity but with an excellent cycle life over 8000 cycles at 1 A g^−1^ (maintained 78%). Shortly, this new macrocycle will suggest a new molecule design for ALB cathode materials.

In spite of the advantages of other materials applied in ZIBs, macrocycles have attracted much interest in ZIBs. Recently, Song et al. [155] used tetranitroporphyrin (TNP) macrocycles as cathode materials in ZIBs. Notably, a TNP macrocycle molecule has four nitro groups and two amine groups which integrate n-type and p-type features, leading to a bipolar macrocycle. The four nitro groups can couple four Zn^2+^ with 8e^−^ conversion and two amine groups couple two SO_4_^2−^ with 2e^−^ conversion, resulting in a 10 charge storage in the discharge process (Figure 18e), which results in the TNP delivering a discharge capacity of 338 mAh g^−1^ at 0.2 A g^−1^ and a compromising voltage of 1.08 V between n-type and p-type cathode materials. The global *π*-conjugated feature of the TNP macrocycle largely prevents the dissolution of the TNP in electrolytes and improves the stability (maintained 98.3% of capacity after 5000 cycles).

We summarize the electrochemical performance of these novel macrocycles in EES/EEC in Table 4. In summary, these novel macrocycles have shown tremendous promising applications in EES/EEC, but extensive efforts are required to improve the electrochemical performance of these macrocycles for commercial application compared with LIBs. This puts forward the idea of more subtle molecular designs for macrocycles used as electrode materials. The research on the above novel macrocycles has demonstrated that a multielectron storage mechanism is favorable for the improvement of the specific capacity of various batteries, which may be a practical strategy for novel macrocyclic molecule design.

## 7. Limitation and Prospects for Macrocycles in Electrochemical Energy Storage and Conversion

Although these star macrocycles (CDs, CAs, CBs, and PAs) have shown promising applications in batteries and supercapacitors as electrodes, electrolytes, binders, and separators, studies on macrocycles for electrochemical energy storage and conversion are still limited in the laboratory. The major limitations of these macrocycles are summarized as below:

**High solubility**: The main components based on organic macrocycles have proven to be soluble in conventional battery electrolytes, and many strategies were reported to prevent the macrocycles dissolving in electrolytes. However, there are many other issues along with these strategies which can decrease the capacity and stability of batteries and supercapacitors.

**Low cycling life**: A longer cycling life is a key consideration of EES/EEC devices used in practical application. The current macrocycle-based batteries and supercapacitors have a sharp reduction in capacity compared with commercial LIBs.

**Economic applicability:** Another key consideration is the cost of macrocycle-based battery materials. As reported, the composites based on macrocycles were combined with graphene, nanomaterials, and polymers, which increased the cost of macrocycle-based batteries and supercapacitors. Moreover, there is a need to decrease the cost of macrocycles and explore more cost-effective approaches for their synthesis.

To overcome these limitations, it is suggested that future studies focus on the following: (a) exploring novel strategies to improve their structural and physical characteristics, leading to an increase in capacity as well as cycling stability; (b) disclosing the mechanisms of the interactions between these macrocycles and modified functional materials, cell components, and metal ions to favor the design of better batteries; (c) finding economic materials and exploring facile methods to reduce the cost of the production process; (d) reducing the environmental impacts of the whole process, including the production, use, and recycling induced by macrocycle-based devices.

## 8. Conclusions

In this review, the recent progress of macrocycle-based EES/EEC devices has been summarized. The current successes suggest that macrocycles show promise in improving the electrochemical performance of LIBs, LSBs, SIBs, supercapacitors, etc. Due to the advantages of cavity structures, hydroxyl groups, carbonyl groups, and easy functionalization, these macrocycles can be used as binders, electrodes, electrolytes, and separators after being modified and/or composited with other functional materials, in terms of providing an ion diffusion channel, integrating electrode materials and redox-active sites, resulting in higher capacity, energy density, and longer cycling life. In spite of the successes, more efforts are needed to improve the performance of these macrocycle-based EES/EEC devices and make them competitive for practical application compared with commercial LIBs.

## Figures and Tables

**Table 1:** The application of CDs in EES/EEC devices.

Application	Devices	Synthesis	Specific Capacity or Capacitance	Working Voltage	Cycle Life (Cycle)	Advantage/ Disadvantage	Ref.
β-CDp/binder	LIBs	Polymerization	~1600 mAh g^−1^	0–1.0 V	150 (retention 90%)	Strong crosslinking/low rate	[80]
PR-PAA/binder	LIBs	Polymerization	2971 mAh g−1		50 (retention 98%)	High reversibility/complicated synthesis	[82]
C-β-CD/binder	LIBs	Oxidation	982 mAh g^−1^	0.02–3 V	200 (retention 626 mAh g^−1^)	Improving rate/poor cycle life	[88]
β-CD/channel	LIBs	Adsorption	2562 mAh g^−1^	0.01–1.5 V	200 (retention 1944 mAh g^−1^)	Promoting Li^+^ transport/poor cycle life	[89]
β-CD/template	ARLBs	Sol–gel route	189 mAh g^−1^	0.5–1.4 V	100 (retention 36%)	High porous structure/poor cycle life	[68]
β-CD/template	LIBs	Hydrothermal synthesis	94 mAh g^−1^	1.8–2.6 V	30 (retention 99%)	High stability/low capacity	[90]
α-CD/template	LISs	Hydrothermal synthesis	700 mAh g^−1^	0.005–3 V	50 (retention < 80%)	High rate/poor cycle life	[91]
β-CD-NH_4_V_4_O_10_/template	LIBs	Hydrothermal synthesis	200 mAh g^−1^	2–4 V	200 (retention 64.9%)	High Li^+^ transition, 3D porous/poor cycle life	[95]
poly-CDQA/adsorption	LSBs	Melt diffusion	1307 mAh g^−1^	1.5–3.0 V	100 (retention 84%)	High adsorption of polysulfides/poor cycle life	[96]
rGO@β-CDP@S/receptor	LSBs	Crosslinking	1329 mAh g^−1^	1.7–2.8 V	300 (retention 85.8%)	Host–guest interaction/complicated synthesis	[97]
C-β-CD/binder	LSBs	Oxidation	1542.7 mA h g_(sulfur)_^−1^	1–3 V	50 (retention 1456 mA h g_(sulfur)_^−1^)	Strong bonding strength/high decay rate	[98]
β-CDp-N^+^/binder	LSBs	Polycondensation	1380 mAh g^−1^	1.7–2.8 V	100 (retention 928 mAh g^−1^)	High initial capacity/high decay rate	[99]
Carbonβ-CD/interlayer	LSBs	Hydrothermal synthesis	~1400 mAh g^−1^	1–3 V	100 (retention 63.8%)	Increased conductivity/high decay rate	[100]
β-CD/template	VFBs	Cast	-	0.8–1.65 V	50 (retention 80% of energy efficiency)	Easily fabrication/high decay rate	[106]
hydroxypropyl-β-CD	AOFBs	Mixing	-	0.3–1.1 V	0.041% per cycle	High solubility/instability	[107]
β-CD polymer/template	ZIBs	Pyrolysis	~800 mAh g^−1^	0–1 V	-	High ECSA/low lifespan	[108]
rGO@β-CDP@PEG-AD/host	Supercapacitors	Mixing	163 F g^−1^	−1 to 0 V	10,000 (retention 80%)	Host–guest interaction/complicated fabrication	[109]
Carbon β-CD/Carbon	Supercapacitors	Hydrothermal reduction	310.8 F g^−1^/at 0.5 A g^−1^	−1 to −0.2 V	10,000 (retention 100%)	Enlarging interlayer spacing/-	[110]
CDP/host	Supercapacitors	Mixing	107.4 F·g^−1^ at 1 A·g^−1^	−0.2 to 0.8 V	5000 (retention 97%)	Guest-recognition capability/complicated fabrication	[111]
PMnCD/template	Supercapacitors	Electrospun, Hydrolysis	228 F g^−1^ at 1 mAcm^−2^	−0.2 to 0.8 V	10,000 (retention 94%)	Hierarchical porous structure/complicated fabrication	[112]

**Table 2:** The applications of CAs in EES/EEC.

Application	Devices	Synthesis	Specific Capacity or Capacitance	Working Voltage	Cycle Life (Cycle)	Advantage/Disadvantage	Ref.
C4Q/cathode	LIBs	Diazocoupling reaction, reduction, oxidation	422 mAh g^−1^	1.5–3.5 V	100 (retention 379 mAh g^−1^)	Coupling 8 Li/high solubility	[69]
C6Q/cathode	LIBs	Diazocoupling reaction, reduction, oxidation	425 mAh g^−1^ at 0.05 C	1.3–3.7 V	500 (retention 405 mAh g^−1^)	High insolubility/poor cycle life at high rate	[117]
C6Q/cathode	LIBs	Diazocoupling reaction, reduction, oxidation	423 mAh g^−1^	1.3–3.7 V	300 (retention 195 mAh g^−1^)	Coupling 12 Li/high solubility in LiPF_6_	[118]
C4Q/cathode	LIBs	Stirred	39 mAh g^−1^	2–4 V	-	Increasing insolubility/losing capacity	[119]
C4Q-CMK-3/cathode	LIBs	Ultrasonicated	427 mAh g^−1^	1.5–3.5 V	100 (retention 58.7%)	High conductivity/poor cycle life	[120]
C4Q-CMK-3/cathode	SIBs	Ultrasonicated	438 mAh g^−1^	1.2–4.2 V	50 (retention 219 mAh g^−1^)	High conductivity/poor cycle life	[123]
C4Q/CMK-3/SWCNTs/cathode	SIBs	Stirring	440 mAh g^−1^	1.2–4.2 V	100 (retention 290 mAh g^−1^)	High conductivity/poor cycle life	[124]
C4Q-[PY13][TFSI]/cathode	SIBs	Diazocoupling reaction, reduction, oxidation	863 Wh kg^−1^ (energy density)	1.2–3.7 V	300 (retention 99.7%)	High cycle life/low density	[126]
C4Q/cathode	AABs	Diazocoupling reaction, reduction, oxidation	400 mAh g^−1^	0.5–1.5 V	50 (retention 81%)	Great capacity/low cycle performance	[129]
C4Q/cathode	ZBs	Diazocoupling reaction, reduction, oxidation	335 mAh g^−1^	0.2–1.75 V	500 (retention 87%)	High capacity/high solubility	[131]
PANI-ES/SC6-MoS_2_/stabilizer	Supercapacitor	Stirring	691 F g^−1^		5000 (retention 91%)	High capacitance/-	[132]

**Table 3:** The applications of PAs in EES/EEC.

Application	Devices	Synthesis	Specific Capacity or Capacitance	Working Voltage	Cycle Life (Cycle)	Advantage/Disadvantage	Ref.
PQ5/cathode	LIBs	Oxidation	418 mAh g^−1^	1.8–3.3 V	50 (retention 94.7%)	High capacity/high solubility	[140]
Poly-P5Q/cathode	LIBs	Polymerization	105 mAh g^−1^	1.75–3.25 V	100 retention 82.3%)	High porous/high solubility	[70]
P5Q/cathode	LIBs	Oxidation	408 mAh g^−1^	1.2–3.9 V	200 (retention 70%)	High capacity/high fade rate	[141]
P5Q-CMK-3/cathode	SIBs	Ultrasonic mixing	418 mAh g^−1^	1.5–4.2 V	300 (retention 69.4%)	High capacity/high fade rate	[142]
RGO-HP5A/working electrode	Supercapacitor	Stirring	331 F·g^−1^ (calculation)	−0.2 to 0.8 V	10,000 (retention 93% at 5 A·g^−1^)	High cycle life/low capacitance	[143]

**Table 4:** The applications of recent novel macrocycles in EES/EEC.

Application	Devices	Synthesis	Specific Capacity or Capacitance	Working Voltage	Cycle Life (Cycle)	Advantage/Disadvantage	Ref.
PMDI-Δ/anode	LIBs	Refluxing in acetic acid	163 mAh g^−1^	1.6–3.2 V	50 (retention 86 mAh g^−1^)	Single output voltage/low cycle life	[151]
PCT/anode	SIBs	Wittig reaction	133 mAh g^−1^	0.01–2 V	500 (retention 100%)	High stability/low capacity	[153]
PQ-Δ/cathode	AIBs	Ni(COD)_2_/2,2′-bipyridine/COD	94 mAh g^−1^	0.7–1.75 V	200 (retention 82 mAh g^−1^)	High stability/low capacity	[152]
TDK/cathode	AIBs	Benzoin condensation	350 mAh g^−1^	0.4–2.1 V	8000 (retention 78%)	High stability/complicate synthesis	[154]
TNP/cathode	ZIBs	BF_3_-Et_2_O,DDQ,Triethylamine	338 mAh g^−1^	0.4–1.7 V	5000 (retention 98.3)	High stability/-	[155]

## Data Availability

All the data presented in this manuscript were derived from the articles indicated, which are published in the literature and listed in the Reference section.

## Abbreviations

EES/EEC	Electrochemical energy storge and conversion
LIBs	Lithium-ion batteries
LSBs	Lithium-S batteries
SIBs	Sodium-ion batteries
MIBs	Magnesium-ion batteries
ZIBs	Zinc-ion batteries
PIBs	Potassium-ion battery
ARLBs	Aqueous rechargeable lithium batteries
AOFBs	Aqueous organic flow batteries
MOFs	Metal organic frameworks
COFs	Covalent organic frameworks
CDs	Cyclodextrins
CAs	Calixarenes
CBs	Cucurbiturils
PAs	Pillararenes
CNTs	Carbon nanotubes
GO	Graphene oxide
RGO	Reduced graphene oxide
β-CDp	β-cyclodextrin polymer
PAA	Poly-(acrylic acid)
EES/EEC	Electrochemical energy storge and conversion
LIBs	Lithium-ion batteries
PR	Pseudo-rotaxanes
C-β-CD	Carbonyl-β-cyclodextrin
poly-CDQA	Poly-β-cyclodextrin quaternary ammonium
rGO@β-CDP@S	Reduced graphene oxide and cyclodextrin polymers and S
VFB	Vanadium redox flow battery
AEM	Anion exchange membrane
HPSf-Im	Hydroxyl polysulfone with imidazolium function alization
HPNSC	Hierarchically porous N and S doped carbon
ECSA	Electrochemically active surface area
PEG-AD	adamantine end-capped poly(ethylene oxide) polymer linker
PMnCD	Porous carbon nanofiber/MnO_2_ composites derived from polyacrylonitrile CD
CQs	Calix[*n*]quinones
C4Q	Calix[4]quinone
PMA/PEG	Poly(methacrylate)/poly(ethylene glycol)
GPE	Gel polymer electrolyte
C6Q	Calix[6]quinone
PCE	Plastic crystal electrolyte
CMK-3	Order mesoporous carbon
IL	Ionic liquid
PEO	Poly(ethylene oxide)
SWCNTs	Single wall carbon nanotubes
[PY13][TFSI]	*N*-methyl-*N*-propylpyrrolidinium bis(trifluoromethanesulfonyl)amide
AABs	Aqueous aluminum batteries
Al(OTF)3	Aluminum trifluoromethanesulfonate
ZBs	Zinc batteries
PANI	Polyaniline
SC6	Sulphatocalix[6]arene
PANI-ES	Sulphatocalix[6]arene doped PANI
CBC	Subnanoporous carbon
MMIMBF4	1-methyl-3-methylimidazole tetrafluoroborate
CPE	Composite polymer electrolyte
P5Q	Pillar[5]quinone
PMA	Poly(methacrylate)
PEG	Poly(ethylene glycol)
POP	Porous organic polymers
SPCC	Supramolecular coordination compounds
DFT	Density functional theory
PMDI-Δ	Triangular pyromellitic diimide
PCT	[2.2.2.2]paracyclophane-1,9,17,25-tetraene
PQ	Phenanthrenequinone
TDK	Tetradiketone
TNP	Tetranitroporphyrin
